# Differential modulation of glioma metabolism and the tumor microenvironment following dexamethasone and bevacizumab treatment

**DOI:** 10.1093/noajnl/vdag147

**Published:** 2026-06-08

**Authors:** Louise Maise, Felix Krautwurst, Surender Surender, Gyuntae Bae, Laimdota Zizmare, Jil Trampert, Marko Maric, Susanne Beck, Marcos Tatagiba, Christoph Trautwein, Hannes Becker, Ghazaleh Tabatabai

**Affiliations:** Department of Neurology & Interdisciplinary Neuro-Oncology, University Hospital Tuebingen, Hertie Institute for Clinical Brain Research, Eberhard Karls University Tuebingen, Tuebingen, Germany; Department of Neurology & Interdisciplinary Neuro-Oncology, University Hospital Tuebingen, Hertie Institute for Clinical Brain Research, Eberhard Karls University Tuebingen, Tuebingen, Germany; Department of Neurology & Interdisciplinary Neuro-Oncology, University Hospital Tuebingen, Hertie Institute for Clinical Brain Research, Eberhard Karls University Tuebingen, Tuebingen, Germany; Werner Siemens Imaging Center, Department of Preclinical Imaging and Radiopharmacy, Eberhard Karls University Tuebingen, Tuebingen, Germany; Cluster of Excellence (EXC 2180) “Image-Guided and Functionally Instructed Tumor Therapies”, Eberhard Karls University Tuebingen, Tuebingen, Germany; Werner Siemens Imaging Center, Department of Preclinical Imaging and Radiopharmacy, Eberhard Karls University Tuebingen, Tuebingen, Germany; Cluster of Excellence (EXC 2180) “Image-Guided and Functionally Instructed Tumor Therapies”, Eberhard Karls University Tuebingen, Tuebingen, Germany; Department of Neurology & Interdisciplinary Neuro-Oncology, University Hospital Tuebingen, Hertie Institute for Clinical Brain Research, Eberhard Karls University Tuebingen, Tuebingen, Germany; Department of Neurology & Interdisciplinary Neuro-Oncology, University Hospital Tuebingen, Hertie Institute for Clinical Brain Research, Eberhard Karls University Tuebingen, Tuebingen, Germany; Department of Neurology & Interdisciplinary Neuro-Oncology, University Hospital Tuebingen, Hertie Institute for Clinical Brain Research, Eberhard Karls University Tuebingen, Tuebingen, Germany; Cluster of Excellence (EXC 2180) “Image-Guided and Functionally Instructed Tumor Therapies”, Eberhard Karls University Tuebingen, Tuebingen, Germany; German Cancer Consortium (DKTK), DKFZ partner site Tuebingen, Eberhard Karls University Tuebingen, Tuebingen, Germany; German Cancer Consortium (DKTK), DKFZ partner site Tuebingen, Eberhard Karls University Tuebingen, Tuebingen, Germany; Department of Neurosurgery, University Hospital Tuebingen, Eberhard Karls University Tuebingen, Tuebingen, Germany; Werner Siemens Imaging Center, Department of Preclinical Imaging and Radiopharmacy, Eberhard Karls University Tuebingen, Tuebingen, Germany; Cluster of Excellence (EXC 2180) “Image-Guided and Functionally Instructed Tumor Therapies”, Eberhard Karls University Tuebingen, Tuebingen, Germany; M3 Research Center for Malignome, Metabolome and Microbiome, Faculty of Medicine, Eberhard Karls University Tuebingen, Tuebingen, Germany; Core Facility Metabolomics, Faculty of Medicine, Eberhard Karls University Tuebingen, Tuebingen, Germany; Department of Neurology & Interdisciplinary Neuro-Oncology, University Hospital Tuebingen, Hertie Institute for Clinical Brain Research, Eberhard Karls University Tuebingen, Tuebingen, Germany; Department of Neurosurgery, University Hospital Tuebingen, Eberhard Karls University Tuebingen, Tuebingen, Germany; Department of Neurology & Interdisciplinary Neuro-Oncology, University Hospital Tuebingen, Hertie Institute for Clinical Brain Research, Eberhard Karls University Tuebingen, Tuebingen, Germany; Cluster of Excellence (EXC 2180) “Image-Guided and Functionally Instructed Tumor Therapies”, Eberhard Karls University Tuebingen, Tuebingen, Germany; German Cancer Consortium (DKTK), DKFZ partner site Tuebingen, Eberhard Karls University Tuebingen, Tuebingen, Germany

**Keywords:** bevacizumab, dexamethasone, glioblastoma, immunometabolism, tumor microenvironment

## Abstract

**Background:**

Dexamethasone (DEXA) is the routine therapy for tumor- or treatment-associated edema management in glioblastoma, whereas bevacizumab (BEV) is increasingly used as a steroid-sparing alternative. Although both reduce edema, their broader immunometabolic effects remain ill-defined. Here, we examine how DEXA and BEV differentially affect tumor metabolism and microenvironment in patient samples and experimental models.

**Methods:**

We integrated ^1^H-NMR-based metabolomics of human glioblastoma specimens with mechanistic *in vitro* studies to compare DEXA and BEV. Microenvironmental modulation by DEXA vs BEV was further investigated *in vivo* in a syngeneic, immunocompetent orthotopic glioma mouse model by flow cytometry and immunohistochemistry, followed by *ex vivo* co-culture models.

**Results:**

Tumors from DEXA-treated patients (*n* = 12) vs steroid-naive controls (*n* = 18) showed nine significantly altered metabolites, including increased lactate, cystathionine, and 2-hydroxybutyrate, indicating a metabolically accelerated, proliferation-associated state. In an immunocompetent orthotopic glioma model, DEXA reduced intratumoral T cell infiltration and induced cytokine conditions favoring regulatory T cells (T_regs_) and myeloid recruitment. In contrast, BEV elicited a coordinated immunostimulatory phenotype: it increased chemotactic cytokines *in vitro* (eg CCL5), decreased intratumoral T_regs_ (CD4^+^FOXP3^+^), enhanced activated, Granzyme B (GzmB) expressing effector T cells (CD4^+^GzmB^+^) *in vivo*, and improved spleenocyte-mediated tumor cell killing *ex vivo*.

**Conclusions:**

Together, DEXA promotes an immunosuppressive, metabolically active tumor microenvironment, whereas BEV supports immune infiltration and activation. These data, combining tissue-derived metabolomics with functional and mechanistic studies *in vitro*, *ex vivo*, and *in vivo*, reveal fundamentally divergent immunometabolic effects of anti-edematous therapies with direct implications in clinical practice, particularly alongside immunotherapy in glioblastoma.

## Graphical Abstract

**Graphical Abstract:**
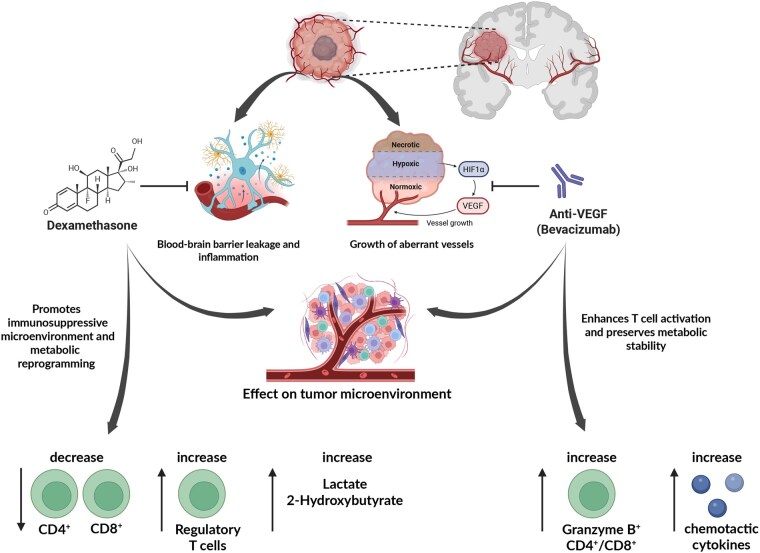

Key Points

- DEXA induces distinct metabolic shifts in glioblastoma.
- DEXA promotes an immunosuppressive microenvironment, reduces T cell infiltration and enriches T_regs_  *in vivo*.
- BEV enhances T cell activation and increases chemotactic cytokines.

Importance of the Study

Our study comprehensively profiles the metabolomic effects of DEXA in human glioblastoma tissue, revealing increased lactate and 2-hydroxybutyrate and decreased adenosine and glucose. Unlike BEV, DEXA fosters an immunosuppressive tumor microenvironment, reducing T cell infiltration and enriching T_regs_ in preclinical glioma models. In contrast, BEV minimally alters metabolism while enhancing T cell activation and reducing T_reg_ proportions.

These findings suggest that DEXA’s immunosuppressive effects might impair clinical trials evaluating immunotherapies for glioblastoma. These data support the strategy to use BEV as an alternative to DEXA for edema management to preserve or even enhance antitumor immune responses, with further studies needed to validate these findings.

Diffuse gliomas, particularly Glioblastoma CNS WHO grade 4, remain among the most aggressive and treatment-refractory solid tumors.^1^ Despite advances in therapeutic modalities, under the current standard of care - maximal safe resection followed by radiotherapy with concomitant and adjuvant temozolomide, with or without tumor-treating fields - median overall survival (OS) for patients remains in the range of 15-18 months, even in selected clinical trial populations.^2^

Glucocorticoids - particularly Dexamethasone (DEXA) - are routinely used to manage peritumoral cerebral edema in neuro-oncology, although some guidelines advise against its use in asymptomatic patients.^1^ Edema arises from therapy-associated necrosis, inflammation, and tumor-secreted vascular endothelial growth factor A (VEGFA), which promotes leaky vasculature.^3^^,^^4^ Long-term DEXA use is associated with adverse outcomes and may shorten OS in glioblastoma patients.^5-7^ DEXA reduces the benefit of immune checkpoint-inhibitors (ICI) in preclinical models, and its administration of ≥3 mg/day at the time of randomization has been listed as an exclusion criterion in numerous clinical studies of ICIs in glioblastoma.^8^ Besides its use as an anti-angiogenic drug in metastatic solid tumor entities, Bevacizumab (BEV), a monoclonal antibody targeting VEGF, also presents an alternative approach to managing cerebral edema. BEV is particularly applied in the context of radiation-induced edema (e.g. PRIDE, NOA-28; ARO-2022-12; NCT05871021).^9^ BEV is used selectively for symptomatic relief, often as a steroid-sparing option, as well as in the context of immunotherapy-associated edema in clinical trials.^10^^,^^11^ This clinical application of BEV is different from its use as anti-tumoral therapy. BEV is not approved for progressive glioblastoma in all countries (eg not approved in the European Union), and although it improved progression-free survival (PFS), it did not demonstrate an OS benefit.^12^^,^^13^

However, the clinical decision between DEXA and BEV for the treatment of tumor- and/or treatment-associated edema currently varies, even in clinical trial protocols: while DEXA is permitted as a co-medication in some clinical trials (including immunotherapy trials, e.g. to treat immunotherapy-associated adverse events), it is explicitly excluded in other study protocols. Similarly, BEV is sometimes prohibited depending on study design and therapeutic context. This heterogeneity might reflect the lack of mechanistic insight into how DEXA and BEV differentially influence tumor metabolism, the immune microenvironment, and therapeutic response. In fact, our current molecular understanding of their effects remains limited. While DEXA partially restores blood-brain barrier integrity via upregulation of tight junction proteins, its broader effects on the metabolic and immunological features of the glioma microenvironment remain poorly defined.^5^ Furthermore, BEV-induced effects remain to be elucidated.

We aimed at addressing this research gap and investigated metabolic and immunological effects following DEXA vs BEV. Our study incorporates comprehensive tissue-based metabolomic profiling, as well as mechanistic and functional investigations *in vitro*, *in vivo*, and *ex vivo*. We identified distinct features of both treatments on tumor metabolism and the glioma-associated immune microenvironment. Our results might inform clinical practice and study designs for clinical trials, particularly trials investigating immunotherapeutic approaches.

## Methods

### Study Cohort and Comparisons Based on Clinical Characteristics

**Figure 1.**
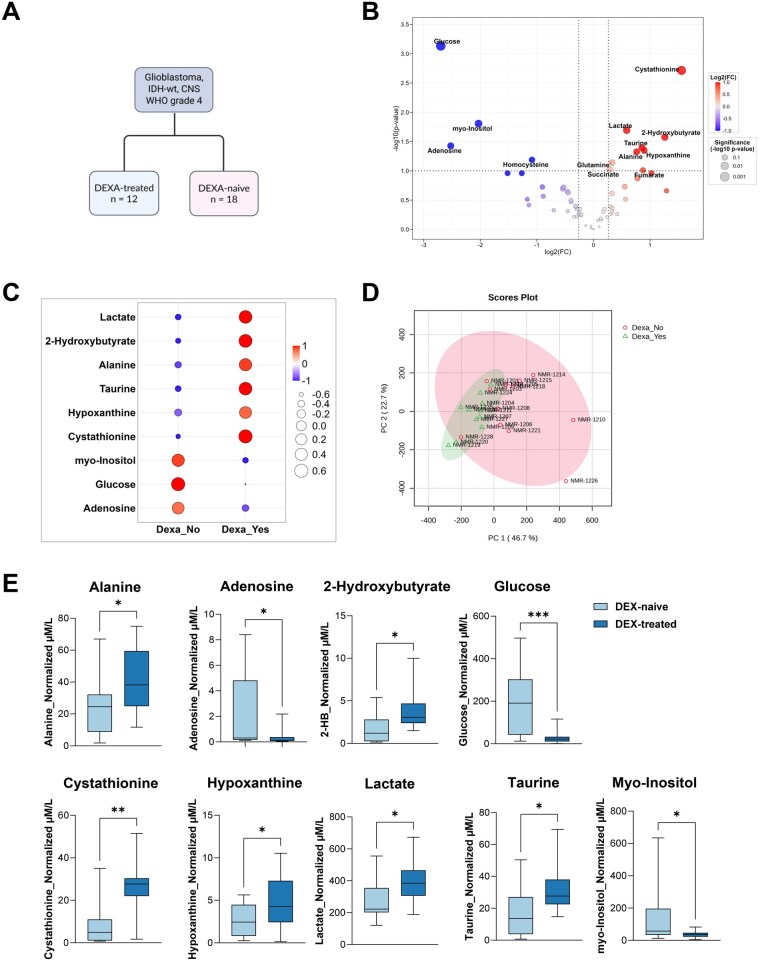
DEXA modulates metabolites in human glioma tissue analyzed by ^1^H-NMR spectroscopy. (A) Study cohort overview showing 18 steroid-naïve patients (*n* = 18) and 12 DEXA-treated patients (*n* = 12). Created in BioRender. Walter, B. (2026) https://BioRender.com/43gut7j (B) Volcano plot illustrating significant metabolic changes under DEXA treatment conditions; horizontal dashed line, *P* = .1; vertical dashed lines, fold change ± 1.2. Nine metabolites exceed thresholds: increased alanine, cystathionine, 2-hydroxybutyrate, hypoxanthine, lactate, taurine; decreased adenosine, glucose, myo-inositol. (C) Bubble plots of the nine significantly changed metabolites; bubble size, normalized average group difference; y-axis, -log_10_(P-value) from Wilcoxon rank-sum test; x-axis, log_2_ (fold change). (D) Principal component analysis (PCA) scores plot (PC1 vs PC2); DEXA-treated and steroid-naïve samples; PC1=56.8%, PC2=15.2%. (E) Box-and-whisker plots (median, quartiles, min-max) of normalized concentrations (arbitrary units) for the nine significantly changed metabolites; unpaired t-test, *P < .05, **P < .01, ***P < .001. Data as mean ± SEM.

The study was approved by the ethical board of the University Hospital Tübingen (reference no. 484/2016BO1). We retrospectively included 30 patients treated at the University Hospital Tübingen between 2014 and 2017 with archival high-quality tissue samples and a confirmed diagnosis of Glioblastoma, WHO CNS grade 4 as well as written informed consent for biobanking of tissue.^14^ Integrated histological diagnosis included IDH1 status, 1p/19q codeletion status, ATRX and promoter methylation status of *MGMT*. This retrospective patient cohort was stratified by glucocorticoid treatment (Figure 1A). We identified 12 patients which had received DEXA treatment (4-32 mg daily) in the 2 weeks prior to surgery and tissue acquisition. In these patients, indication for DEXA administration was based on the presence of MRI-confirmed large perifocal cerebral edema associated with clinical symptoms, reflecting standard clinical practice. Furthermore, all patients received one DEXA infusion (20 mg) perioperatively as part of the routine neurosurgical procedure. Clinical characteristics of the patient cohort are summarized in Supplementary Tables S1 and S2.

### Immuno-Oncology Panel

We prepared tumor tissue lysates from samples of the described patient cohort (Figure 1A). These lysates were analyzed in an Immuno-Oncology panel including 92 proteins (OLINK, Uppsala, SE) in accordance with the manufacturer’s guidelines. In brief, tumor tissue lysates were prepared with RIPA buffer (Merck), 1 mM phenyl-methane-sulfonyl fluoride (PMSF) (Sigma-Aldrich) and 1 tablet protease inhibitor cocktail complete (Roche).

### Metabolite Extraction from Archival Biological Samples

Fresh resection specimens of the described patient cohort (Figure 1A) were immediately snap-frozen in liquid nitrogen and stored at -80°C prior to long-term archival storage in good accordance to proposed framework of glioma tissue sampling by Response Assessment in Neuro-Oncology (RANO) working groups.^15^^,^^16^ Deep-frozen biopsy samples (50-100 mg) were cryogenically pulverized (cryoPREP, Covaris Inc.) and lyophilized overnight (Alpha 2-4, Martin Christ). 5 mg of lyophilized glioma powder were transferred into 2 mL adaptive focus acoustics (AFA) glass tubes (milliTUBE, Covaris Inc.) and extracted with 400 µL ultrapure methanol (MeOH) and 800 µL methyl tert-butyl ether (MTBE, solvent grade). Homogenization and metabolite extraction were performed in a degassed water bath at 7°C using a focused ultrasonicator (E220evolution, Covaris Inc.), applying two consecutive sonication programs with vertical sample movement (5 min each). Phase separation was induced by adding 600 µL ultrapure water, followed by centrifugation at 5,000 g for 5 min at 4°C. Polar phase was collected and evaporated to dryness overnight using a vacuum concentrator (Concentrator 5310, Eppendorf). Dried extracts were resuspended in deuterated phosphate buffer (200 mM K_2_HPO_4_, 200 µM NaN_3_, pH 7.4) containing 1 mM trimethylsilylpropanoic acid (TSP) as internal standard. Samples were vortexed thoroughly and centrifuged at 14,000 g for 5 min at 4°C. Supernatants were transferred into 5 mm or 1.7 mm NMR tubes and placed in a cooled (4°C) autosampler.

### Cell Lines and Reagents

Human glioma long-term cell line LN229 was obtained from ATCC (Wesel, DE), murine glioma cell line GL261 was obtained from DSMZ (Wesel, DE). Murine glioma cell line SMA560 was kindly provided by Prof. Dr. Wick (University Hospital and DKFZ Heidelberg, DE).

All cell lines were cultured in Dulbecco’s Modified Eagle Medium (DMEM, Gibco) supplemented with 10% fetal calf serum (FCS, Thermo Fisher Scientific) and 50 µg/mL Gentamycin (Thermo Fisher Scientific) at 37°C and 5% CO_2_. For serum-free medium (SFM), FCS was omitted. All cell lines were repetitively authenticated by Short Tandem Repeat (STR) profiling at the Leibniz Institute, DSMZ-German Collection of Microorganisms and Cell Cultures GmbH, Braunschweig, DE and tested in-house for negative mycoplasma contamination.

### Acute Cytotoxicity Assay

Assays were performed as previously described.^17^ We seeded cells one day before treatment. Cells were treated for 72 h in SFM with DEXA D4902 (Sigma-Aldrich) or MeOH control at indicated concentrations. Cells were treated in the same manner with BEV (HYP-P9906, MedChemExpress) or human IgG isotype control (#31154, Invitrogen, Thermo Fisher Scientific). Murine glioma cells were treated with anti-VEGF antibody B20-4.1.1 (Genentech) or mouse IgG2a isotype control (BP0085, Biozol) at indicated concentrations. Cell viability was detected on a GloMax upon addition of CellTiterBlue reagent (Promega).

### Clonogenic Survival Assay

Assays were performed as previously described.^17^ We seeded cells one day before treatment at specified concentrations of DEXA D4902 (Sigma-Aldrich) or MeOH in SFM for 24 h. We then incubated cells for a period ranging from 14-21 days in DMEM complete. We stained colonies with crystal violet solution and counted them using an eCount colony counter pen (Heathrow Scientific). Assays were performed in the same manner for LN229 cells using BEV (HYP-P9906) or IgG control (#31154). Measurements were normalized to respective vehicle control wells.

### Preparation of LN229 Cell Extracts for ^1^H-Nuclear Magnetic Resonance Spectroscopy-Based Metabolomics

1 × 10^6^ LN229 cells were cultured in 10 cm petri dishes and treated with four different conditions: DEXA (D4902), BEV (HYP-P9906), IgG control (#31154), and MeOH control at indicated concentrations: 10 μg/mL for BEV and IgG control and 1 µM for DEXA and MeOH control. Cells were harvested after 24 h of treatment, with 5 biological replicates, each containing at least one million cells. For metabolite quenching, cells were washed twice with 5 mL of ice-cold (4°C) Phosphate-Buffered Saline (PBS). Immediately after washing, PBS was removed, and 5 mL of liquid nitrogen were added to each dish. Following evaporation of liquid nitrogen, dishes were placed on dry ice. Cells were scraped in 1.4 mL of ultrapure MeOH pre-chilled to -80°C and transferred to 2 mL milliTUBES (Covaris Adaptive Focused Acoustics AFA). Samples were stored at -80°C.

### Metabolite Extraction from LN229 Cells

Cell pellets were collected, washed with PBS buffer, and quenched in 100 µL ice-cooled ultra-pure MeOH immediately after harvesting (total processing time from quenching to extraction start < 5 min). Suspension was transferred to 2 mL glass tubes (Covaris Adaptive Focused Acoustics AFA), 900 µL of chloroform and 100 µL of ultra-pure water were added, suspensions well mixed, and finally submitted to AFA ultrasound metabolite extraction protocol (Covaris E220 Evolution). Ultrasonication was carried out in a cooled water bath (5.0 to 15.0°C). Each cycle was repeated 5 times, and total run time per sample was 5 min. Following extraction, the mixture was centrifuged at 12,000 g for 10 min and transferred to 2 mL high-performance liquid chromatography glass vials. Samples were concentrated by evaporation to dryness by a vacuum concentrator (Thermo Fisher Speedvac). Pellets were re-suspended in 45 µL 1M K_2_HPO_4_ buffer (pH = 7.4, containing NaN_3_ and 1mM internal NMR standard TSP), thoroughly mixed and centrifuged for 5 min at 30,000 g. 40 µL of supernatant was filled into 1.7 mm NMR tubes.

### ^1^H-NMR Spectra Acquisition

NMR spectra were acquired on a 14.10 Tesla (600 MHz) ultra-shielded NMR spectrometer (Avance III HD, Bruker BioSpin) equipped with 5 mm or 1.7 mm triple-resonance (^1^H, ^13^C, ^15^N/^31^P) room temperature probes. Spectra were recorded at 298 K. A Carr-Purcell-Meiboom-Gill (CPMG) experiment was used for sample spectra acquisition to suppress residual background signals from remaining macromolecules in the solution and water (time domain = 64k points, sweep width = 20 ppm, 2024 scans, 4 h per sample).

### Cytokine Arrays

LN229 cells were seeded one day before treatment for 72 h with 1 µM solution of DEXA (D4902) or MeOH control in SFM. Alternatively, cells were treated with 10 µg/mL BEV (HYP-P9906) or IgG control (#31154). Medium was replaced with culture medium and cells were incubated for further 48 h. Resulting cell-conditioned medium (CCM) was centrifuged at 4,000 g in Amicon Ultra-15 Centrifugal Filter Devices (Merck Millipore) for 30 min. Filtered residue was used to perform ELISA-based cytokine detection assays (ab133996—Human Cytokine Antibody Array Kit, Abcam) according to manufacturer’s instructions. Membrane imaging was performed using the ChemiDoc Imaging System and ImageLab software (Bio-Rad). Analysis was conducted using ImageJ software and the publicly available macro “Protein Array Analyzer.”^18^ Signal normalization was carried out according to protocol provided by the supplier (Abcam).

### Orthotopic Murine Glioma Model

Animals were kept and bred in the institutional animal facility under controlled environmental conditions (12 h light/dark cycle, 20°C-24°C, 40%-60% humidity) with *ad libitum* access to chow and water and environmental enrichment. Up to 5 mice were housed per cage. All experiments were approved by the Tübingen regional council (Regierungspräsidium Tübingen; license N14/23G) and conducted in July-August 2024 in accordance with animal welfare legislation. Experimental design and procedures were predefined in the approved license. Mice were randomized to treatment arms (*n *= 8 per group; total *n* = 32) considering sex, weight, and age; allocation was not concealed and no additional measures were taken to control for cage position or measurement order. Group size was based on experience from previous experiments using the model, as described in the approved animal license.

Tumor cell implantation (10 × 10³ SMA560 cells into the right striatum of 8-12-week-old VM/Dk mice, male and female) was performed under deep 3-component anesthesia (Fentanyl 0.05 mg/kg, Midazolam 5.0 mg/kg, Medetomidine 0.5 mg/kg; s.c.) with postoperative reversal (Flumazenil 0.5 mg/kg, Atipamezole, 2.5 mg/kg, Butorphanol 4 mg/kg; s.c.).^17^ Analgesia (paracetamol 0.2 mg/g KG in drinking water) was provided from 24 h before surgery until 2 days postoperatively. Animals were monitored daily by trained personnel for welfare (Supplementary Table S3).^17^ Due to rapid tumor progression, treatment duration was limited to symptom-free window. No aggressive behavior or mutual injuries among males were observed during the experimental observation period of 16 days. Anti-VEGF antibody B20-4.1.1 (0.5 mg/kg), DEXA (Dexa ratiopharm, ratiopharm GmbH; 0.01 mg/kg) or respective control treatments (NaCl 0.9%, IgG2a BP0085) were administered by i.p. injection for 5 consecutive days with a treatment holiday of 2 days, to model typical treatment schedule of patients (Figure 3A). Humane endpoints were predefined and no animals required euthanasia due to severe postoperative complications or tumor-related symptoms. The experiment was terminated for all groups when the first animal reached endpoint criteria (Supplementary Table S3) on day 16. Final anesthesia was performed by i.p. injection with Ketamine (120 mg/kg) and Xylazine (10 mg/kg) followed by transcardial PBS perfusion and brain extraction. No data were excluded from analysis.

### Extraction of Spleenocytes from Murine Glioma Models

Fresh spleens were removed from SMA560-tumor bearing VM/Dk mice, processed with ice cold PBS and filtered through 100 µm cell strainers. After centrifugation at 1200 rpm for 10 min at 4°C, resulting pellets were resuspended in red blood cell lysis buffer at room temperature for 4 min. RPMI medium was added to stop the reaction, followed by a second centrifugation step. Spleenocytes were cultured in RPMI supplemented with 10% FCS (Thermo Fisher Scientific) and 1% Penicillin-Streptomycin (100X, Thermo Fisher Scientific). For expansion of T cells, we cultured spleenocytes in RPMI containing Glutamine (200 mM; Thermo Fisher Scientific), 10% FCS and 1% Penicillin-Streptomycin supplemented with IL2, IL15, and IL21 (R&D systems).

### Co-Culture of Spleenocytes and Murine Glioma Cell Lines

SMA560 cells were seeded one day before treatment in 96-well plates at 5 × 10^3^ cells per well and 50 × 10^3^ cells per T25 flask. Spleenocytes were added in a 5:1 ratio with either 10 µM DEXA (Dexa-ratiopharm), 10 µg/mL anti-VEGF (B20.4.1.1), 10 µg/mL IgG2a (BP0085) or 10 µM NaCl control. After 72 h coculture time, cell viability was detected on a GloMax upon addition of CellTiterBlue reagent (Promega). For flow cytometry analysis, aspirated medium (containing spleenocytes) was saved. Tumor cells were detached using Accutase (Thermo Fisher Scientific).

### H&E Staining

Brains (*n* = 3 per treatment group) were snap-frozen and embedded in O.C.T. Tissue-Tek (Sakura Finetek Europe B.V.) and cut to 8 µm sections using a cryostat (Leica).

### Immunohistochemistry

BLOXALL Solution (Vector Laboratories) was used to quench internal peroxidase activity. Primary antibodies (CD3, CD4, CD8, CD11b, CD31, Carbonic Anhydrase IX) were incubated overnight at 4°C.^3^ Secondary antibodies were added for 1 h at room temperature. Antibody information is listed in the Supplementary Materials. Vectastain ELITE ABC Reagent kit (Vector Laboratories) was used according to manufacturer’s instructions in combination with NOVA RED (Vector Laboratories) peroxidase substrate. Slides were counterstained with Hematoxylin (Sigma-Aldrich), dehydrated, and mounted in DPX medium (VWR).

### Microscopy and Analysis of IHC Staining

IHC staining was evaluated and captured using an Axiofluor Zeiss microscope (Carl Zeiss) and Axio Vision 4.0 software (Carl Zeiss). Analysis was performed using ImageJ software, as previously described.^19^

### Flow Cytometry

Tumors were macro-dissected from tumor-bearing murine brains (*n* = 3 per treatment group), finely processed in ice-cold DMEM using a sterile blade, and enzymatically digested with Collagenase IV (Sigma-Aldrich) at 37°C for 30 min. After centrifugation at 440 g for 5 min at 4°C, supernatant was removed. Pellet was resuspended in RMPI with 10% FCS and 1% Penicillin-Streptomycin (Thermo Fisher Scientific) and strained through a 70 µm strainer, followed by centrifugation at 440 g for 5 min at 4°C. Resulting pellet was resuspended in 30% Percoll solution (Sigma-Aldrich). Through a Pasteur pipette, 70% Percoll solution was added below the phase of 30% Percoll solution. Samples were centrifuged at 440 g for 20 min at room temperature. Myelin debris was removed from the 70% Percoll layer. A 3 mL of medium from the phase boundary were transferred to a new falcon. Flow cytometry buffer (PBS containing 2% FCS and 0.5M EDTA) was added, and samples were centrifuged at 440 g for 5 min at 4°C. Supernatant was removed. Pellet was resuspended with 1 mL of ACK lysis buffer (Sigma-Aldrich) at room temperature for 5 min. 9 mL flow cytometry buffer was added, and after a final centrifugation step, pellet was resuspended with 500 µL of flow cytometry buffer. Cells were stained for CD4, MHC-II, CD3, CD8A, FOXP3, Granzyme B (GzmB), CD274 (PD-L1), CD45, CD25, and CD279 (PD-1) as well as with Fixable viability dye eFluor 450 (65–0863-18, Thermo Fisher Scientific). Antibodies are listed in the Supplementary Materials. Data were acquired using MACSQuant analyzer (Miltenyi Biotech) and analyzed using FlowJo (version 10.0, BD Biosciences). Gating strategies are shown in Supplementary Figure S6.

### Statistical Analysis

Statistical analyses were performed using R (v4.4.1), RStudio (2024.09.0 + 375), and GraphPad Prism 10 (GraphPad Software). Unless indicated otherwise, we assumed significance when adjusted *P*-values were below .05.

Recorded free induction decays were Fourier transformed and phase- and baseline-corrected using TopSpin 3.6.1 (Bruker). Metabolite annotation and quantification were performed using Chenomx NMR Suite 9.02 (Chenomx Inc.). Prior to statistical analysis, NMR parameters with more than 80% missing values were removed. Low constant value imputation was applied to the remaining missing data. To make samples and features comparable, all data was normalized by a reference sample (PQN) to account for dilution effects. Univariate statistics, such as Wilcoxon rank, unpaired, and Welch’s t-tests were performed based on the results of variance and normal distribution tests. Of note, *P*-value <.05 was considered significant without Benjamini-Hochberg correction, since two group statistical tests inherently control type I and II errors. The MetaboAnalyst 6.0 web server (R-based online analysis tool, www.metaboanalyst.ca) was used for statistical analysis. Cytokine expression data was transformed into binary variables (“high” vs “low”) using median expression as cutoff values for each cytokine.

## Results

### DEXA Modulates Glioblastoma Tissue Metabolites

We examined 30 tumor tissue samples of Glioblastoma, CNS WHO grade 4 with or without DEXA treatment (Figure 1A, Supplementary Tables S1 and S2). Preoperative DEXA administration was based on clinical indication, and no significant differences in confounding clinical baseline characteristics between groups were observed. Patient characteristics were evenly distributed between groups (Supplementary Tables S1 and S2) with no significant differences in *MGMT* promoter methylation status, age, preoperative Karnofsky Performance Score (KPS), extent of resection, or tumor localization. DEXA intake was not significantly associated with OS or PFS. For patients without (*n* = 18) and with DEXA (*n = *12), respectively, median OS was 18 vs 20 months (HR 1.17, *P* = .69), and median PFS was 10 vs 8 months (HR 1.34, *P* = .48).

^1^H-NMR spectroscopy-based metabolomics revealed significant metabolic alterations in tumor tissue from DEXA-treated compared to steroid-naive Glioblastoma, CNS WHO grade 4 patients. Principal component analysis (PCA) demonstrated robust separation of DEXA-treated and -naive samples, indicating distinct metabolic profiles (Figure 1D, Supplementary Figure S1). We identified 9 significantly altered metabolites (unpaired t-test, *P* < .1 and fold change > 1.2) in DEXA-treated compared to steroid-naive samples, including increased lactate, cystathionine, and 2-hydroxybutyrate and decreased adenosine, glucose, alanine, hypoxanthine, myo-inositol, and taurine (Figure 1B, C, and E). As a proof-of-concept analysis, we observed clear separation of metabolomic data according to *MGMT* promotor methylation status in the PCA and a distinct methylation status-associated metabolic signature. 10 metabolites were significantly altered in unmethylated versus methylated tumor samples, including increased leucine and formate levels (Supplementary Figure S2).

### DEXA-Treated Glioblastoma Tissue Samples Exhibit Reduced T Cell Marker Expression and Elevated Immunosuppressive Cytokines in Human Tissue Samples

We assessed the same glioblastoma tissue samples for cytokine gene expression and immune markers using an immune-oncology panel (Supplementary Figure S3). In DEXA-treated glioblastoma samples, cytokine levels of IL6, IL8, CSF1 and CXCL1 were significantly elevated compared to DEXA-naive tumors (*P < .05 for each). Moreover, DEXA-treated samples exhibited significantly lower CD8A expression (*P < .05), whereas CD4 expression did not significantly differ between groups (*P* = .8). We further dichotomized samples into “high” and “low” expression groups according to the median expression value for each cytokine (Table 1). IL6, IL8, CSF1, and CXCL1 “high” expression was significantly more frequent in the DEXA-treated patient group (*P < .05 for each), whereas Programmed death ligand 1 (PD-L1) “high” expression was significantly less frequent in the DEXA group (*P < .05). VEGFA expression showed no significant differences between groups (*P* = .2).

**Table 1.** Inflammatory and angiogenic marker expression by DEXA intake

		DEXA intake		
Characteristic	*n*	No, *n* = 18^A^	Yes, *n* = 12^A^	*P*-value^B^
IL8 expression	30			.034
High		2 (11%)	6 (50%)	
Low		16 (89%)	6 (50%)	
IL6 expression	30			.034
High		2 (11%)	6 (50%)	
Low		16 (89%)	6 (50%)	
CXCL1 expression	30			.034
High		2 (11%)	6 (50%)	
Low		16 (89%)	6 (50%)	
PD-L1 expression	30			.034
High		2 (11%)	6 (50%)	
Low		16 (89%)	6 (50%)	
VEGFA expression	30			.2
High		3 (17%)	5 (42%)	
Low		15 (83%)	7 (58%)	
CSF1 expression	30			.034
High		2 (11%)	6 (50%)	
Low		16 (89%)	6 (50%)	

A Values are *n* (%).

B Fisher’s exact test. DEXA, Dexamethasone. PD-L1, programmed death-ligand 1. VEGFA, Vascular endothelial growth factor A. This table summarizes the expression levels of key cytokines and immune markers (IL8, IL6, CXCL1, PD-L1, VEGFA, and CSF1) in tumor tissue from 30 glioblastoma patients, stratified by DEXA exposure (No, *n* = 18; Yes, *n* = 12). Expression levels are categorized as High or Low, defined by using median expression value as cutoff value, with data presented as n (%). Statistical comparisons between DEXA-treated and DEXA-naive groups were performed using Fisher’s exact test, with *P*-value indicating the significance of differences in expression levels.

### Differential Effects of DEXA and BEV on Glioma Cell Survival *In Vitro*

We next evaluated DEXA and BEV effects on glioma cell survival *in vitro*. Acute cytotoxicity and clonogenic survival assays were conducted in LN229 (human), as well as GL261 and SMA560 (murine) glioma long-term cell lines. While DEXA induced a dose-dependent reduction in cell viability across cell lines except SMA560 (Supplementary Figure S4E and G), BEV treatment had no measurable impact on cell viability (Supplementary Figure S4F and H). DEXA significantly impaired clonogenic potential in LN229 cells at concentrations ≥ 10 µM (Supplementary Figure S4A and C). In contrast, BEV significantly increased clonogenicity in LN229 cells at concentrations ≥ 1 µM (Supplementary Figure S4B and D). No shifts in cell cycle phases were observed in LN229 under BEV treatment, but VEGFR2 expression under BEV was reduced (*P* = 0.13; Supplementary Figure S4I and J).

### ^1^H-NMR Spectroscopy-Based Metabolomics Reveals DEXA-Dependent Changes of Metabolites Involved in Energy and Cell Growth Metabolism *In Vitro*

We performed ^1^H-NMR-spectroscopy-based metabolomics analysis of LN229 glioma cell extracts following treatment either with DEXA (D4902) or BEV (HYP-P9906) at subtoxic concentrations and respective vehicle control for 24 h. DEXA treatment significantly increased concentrations of choline (**P < .01), a precursor metabolite of cell membrane synthesis while the organic acids acetate (**P < .01) were increased or in the case of formate (*P < .05) decreased compared to vehicle control (Supplementary Figure S5A-C). In contrast, BEV treatment significantly increased O-phosphocholine concentration (*P < .05) compared to control, with no other notable metabolic changes observed (Supplementary Figure S5D).

### DEXA- and BEV-Treated Glioma Cells Exhibit Altered Secretion of Chemotactic Cytokines

**Figure 2.**
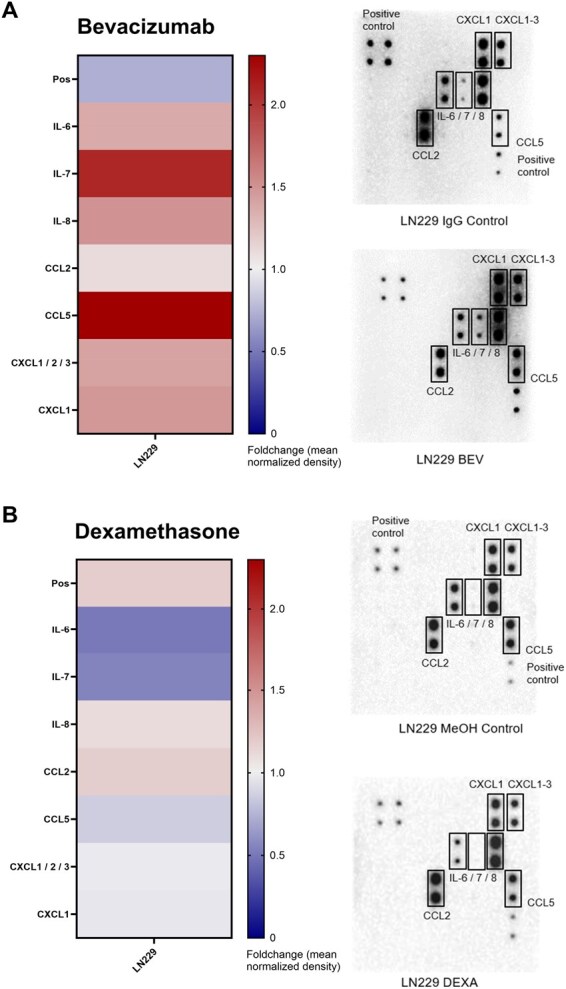
*In vitro* cytokine array analysis of LN229 glioma cells reveals opposing immunomodulatory effects of BEV versus DEXA. (A) Left: Normalized mean cytokine density foldchange in LN229 cells treated for 72 h with BEV versus IgG control. Right: Representative blot images for IgG control (top) and BEV-treated (bottom) cells. BEV treatment increased the abundance of IL6, IL7, IL8, CCL5, and CXCL1/2/3. (B) Left: Normalized mean cytokine density foldchange in LN229 cells treated for 72 h with DEXA versus MeOH vehicle control. Right: Representative blot images from the cytokine array membrane for MeOH control (top) and DEXA-treated (bottom) cells. DEXA treatment significantly reduced the abundance of several pro-inflammatory cytokines, including IL6, IL7, and CCL5. Blots quantified by ImageJ Protein Array Analyzer plugin and normalized to positive controls.

We investigated effects of subtoxic BEV and DEXA treatments on cytokine secretion in LN229 cells *in vitro* by considering normalized signal ratios of ELISA-based cytokine detection membranes. In CCM from DEXA-treated cells, IL6, IL7, and CCL5 levels were reduced, while CCL2 and IL8 showed a moderate increase compared to control (Figure 2B). In contrast, CCM harvested from BEV-treated cells showed broader upregulation of chemotactic and immunomodulatory cytokines in comparison to control, including CCL5, IL6, IL7, IL8, and members of the CXCL1/2/3 family (Figure 2A).

### DEXA Reduces T Cell Infiltration *In Vivo* and Enriches for Regulatory CD8^+^ and CD4^+^ T Cell Subsets in the Immunocompetent VM/Dk-SMA560 Glioma Model

**Figure 3.**
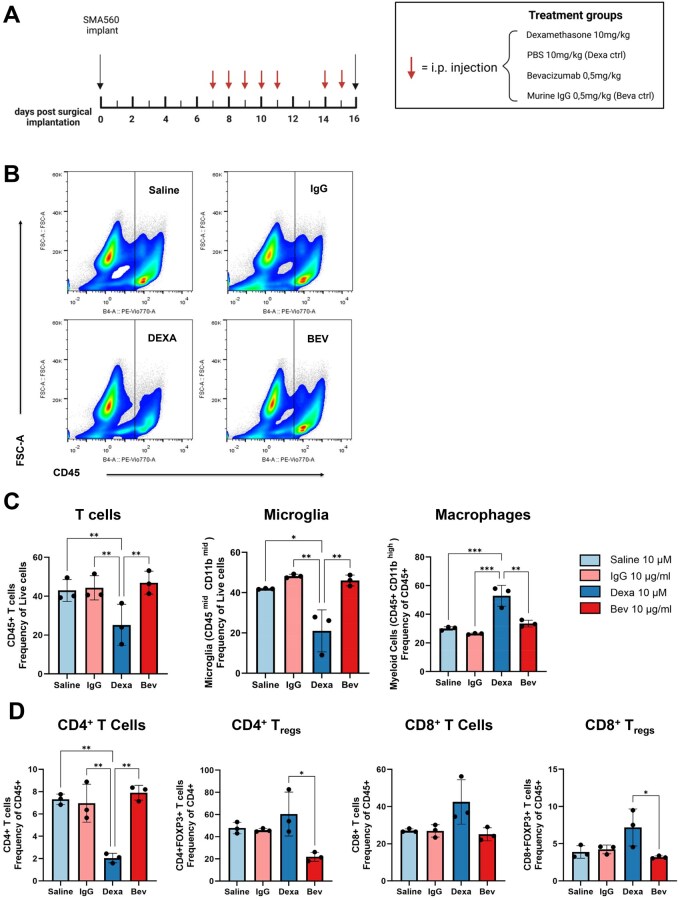
DEXA and BEV modulate the glioma-associated microenvironment in an immunocompetent glioma mouse model. (A) Experimental timeline. SMA560 glioma cells were implanted orthotopically into VM/Dk mice. Intraperitoneal treatments with DEXA, BEV, IgG control, or saline began on day 7. Brains were harvested for analysis on day 16. Created in BioRender. Walter, B. (2026) https://BioRender.com/hbqtrhj (B) FC of CD45^+^ leukocytes (% live cells); DEXA-treated group shows lowest infiltration (C) Among CD45^+^ cells: T cells (CD11b^−^, % CD45^+^); macrophages (CD45ʰⁱᵍʰ CD11bʰⁱᵍʰ, % CD45^+^); microglia (CD45ᵐⁱᵈ CD11bᵐⁱᵈ, % CD45^+^); DEXA reduces T cells, increases macrophages and decreases microglia. (D) T cell subsets (% live cells): total CD4^+^; CD4^+^FOXP3^+^ T_regs_; total CD8^+^; CD8^+^FOXP3^+^ T_regs_; DEXA lowers total CD4^+^ and raises T_reg_ proportions compared to BEV. Data as mean ± SEM, one-way ANOVA, *P <.05, **P <0.01.

We assessed *in vivo* effects of subtoxic DEXA treatment versus BEV treatment with respective controls (NaCl- or IgG2a-treated) in the syngeneic, immunocompetent SMA560-VM/Dk mouse model (Figure 3A). IHC staining for CD31 and Carbonic Anhydrase (CA) IX indicated the presence of hypoxic regions and confirmed the presence of a vascularized tumor microenvironment in all treatment groups (Supplementary Figure S10). We observed a significantly altered tumor-associated immune microenvironment in harvested SMA560 tumors upon DEXA vs BEV and respective control treatments, affecting both the relative frequencies of immune cell populations and their functional state, as reflected by T cell activation and exhaustion (Figure 3B-D, Supplementary Figures S7 and S8).

Flow cytometry analysis revealed significantly lower proportions of live CD45^+^ cells (Figure 3B) and CD45^+^ T cells (Figure 3C) in brains of DEXA-treated mice. DEXA-treated tumors exhibited lower proportions of CD4^+^ T cells among all CD45^+^ cells as compared to NaCl-, IgG2a- and BEV-treated brains (Figure 3D). While CD8^+^ T cell proportions among all CD45^+^ cells increased non-significantly, DEXA treatment significantly enriched for forkhead box P3 (FOXP3) expressing CD8^+^FOXP3^+^ regulatory T cells (T_regs_) among all CD45^+^ cells compared to BEV- and IgG2a-treated brains (Figure 3D). In the subset of CD8^+^ cells, DEXA-treated brains showed significantly higher proportions of CD8^+^FOXP3^+^ CD25^+^ cells as compared to IgG2a- and BEV-treated brains (Supplementary Figure S8B). Similarly, the proportion of CD4^+^FOXP3^+^ T_regs_ among all CD4^+^ cells was significantly higher in DEXA- as compared to BEV-treated mice with BEV-treated brains showing lowest FOXP3^+^ CD4^+^ cell frequencies across all treatment groups (Figure 3D). Lowest FOXP3^+^ CD4^+^ cell frequencies were also observed under BEV treatment among all CD45^+^ cells (Supplementary Figure S8A). Furthermore, tumor-bearing brains from DEXA-treated animals showed a significantly higher proportion of macrophages, defined as CD45^+^CD11b^high^ cells, but reduced proportion of microglia, defined as CD45^mid^CD11b^mid^ cells, as compared to NaCl-, IgG2a-, and BEV-treated brains (Figure 3C).

**Figure 4.**
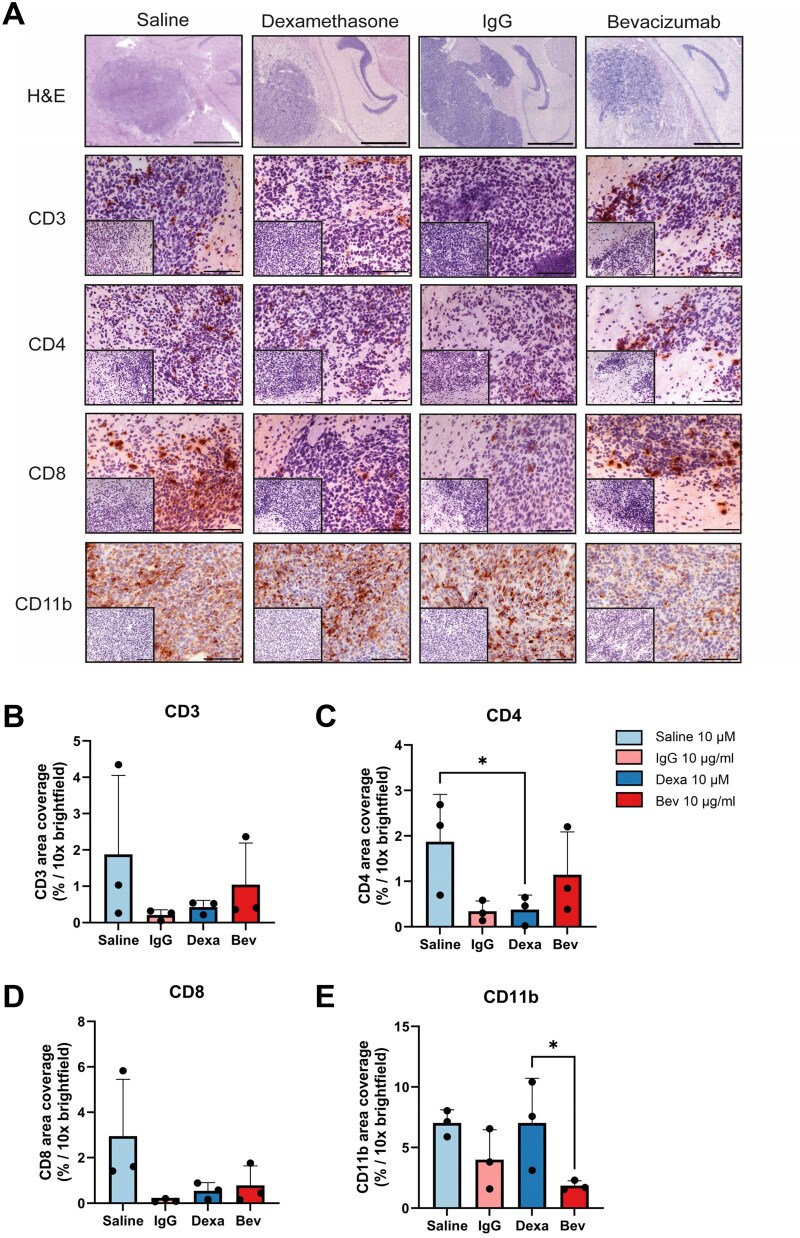
DEXA suppresses T cell infiltration whereas BEV limits CD11b^+^ cell accumulation in glioma. (A) Representative histological and immunohistochemical images of SMA560 glioma tumors from VM/Dk mice treated with saline, DEXA, IgG control, or BEV. Top row: H&E staining (tumor architecture/invasion), 100x magnification, scalebars 2 mm. Rows 2-5: Immunohistochemistry for CD3, CD4, CD8, and CD11b (tumor T cell and myeloid cell infiltration). Insets show secondary antibody control-stained tissue, 200x magnification, scalebars 100 µm. (B–E) Quantification of positively stained area coverage (% 10× brightfield) for CD3 (B), CD4 (C), CD8 (D), and CD11b (E). Data are presented as mean ± SEM. Statistical significance was determined by Kruskal-Wallis test (*P <.05).

IHC for CD3, CD4, CD8, and CD11b was performed on harvested SMA560 tumors (Figure 4A). All treatment groups exhibited stable tumor formation in the right striatum. Robust T cell marker staining was detected at tumor margins across all groups. DEXA treatment resulted in diminished CD3^+^, CD4^+^, and CD8^+^ signals relative to NaCl controls, whereas BEV treatment increased these signals compared with IgG2a controls (Figure 4A-D). Quantification confirmed significantly reduced CD4^+^-positive area in DEXA- versus NaCl-treated tumors (Figure 4C, *P < .05). CD11b-positive areas were larger in DEXA- and NaCl-treated tumors than in BEV- or IgG2a-treated tumors. BEV-treated tumors displayed the lowest CD11b-positive area, significantly lower than in DEXA-treated tumors (Figure 4E), indicating reduced tumor-associated macrophage abundance following BEV treatment.

### BEV Treatment Enhances T Cell Activation Markers in Comparison With DEXA in the Immunocompetent VM/Dk-SMA560 Glioma Model

**Figure 5.**
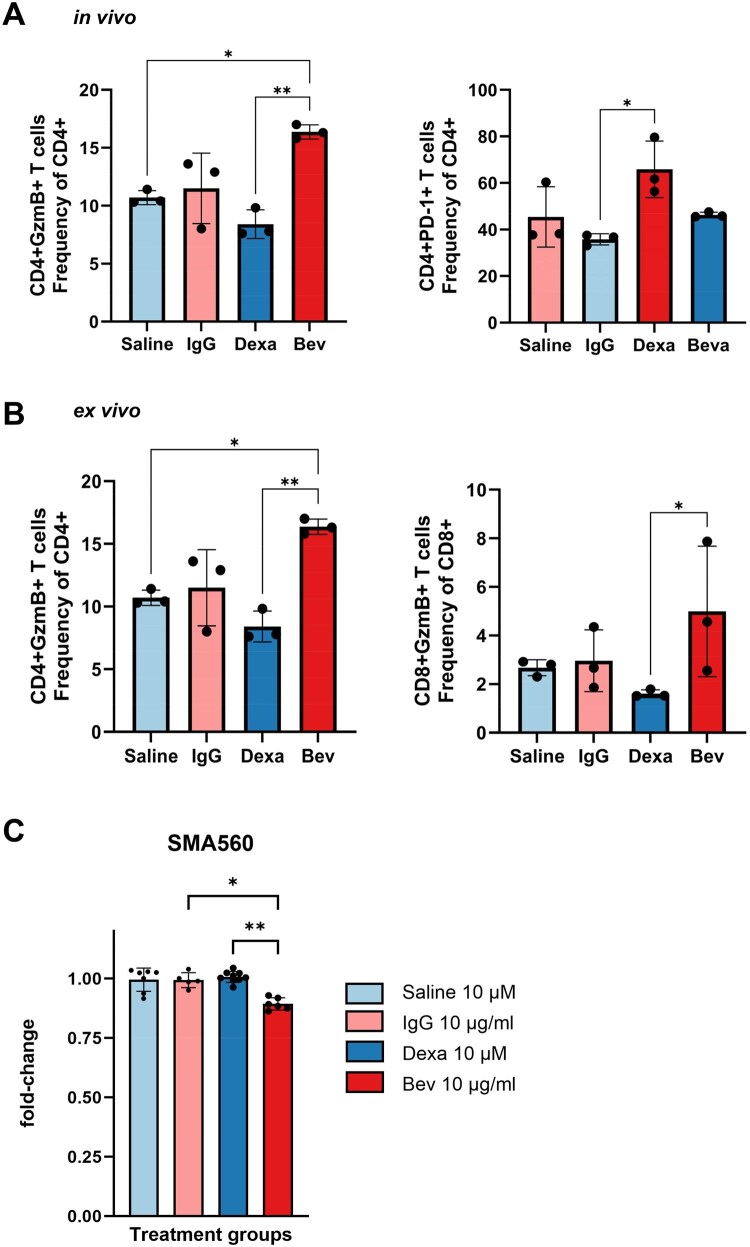
BEV- treatment leads to increased T cell mediated tumor cell killing in an *ex vivo* SMA560-VM/Dk co-culture models. (A) Quantification of tumor-infiltrating immune cells in tumors from syngeneic SMA560-Vm/Dk glioma models by flow cytometry (FC). DEXA treatment significantly increased the frequency of total CD4^+^PD1^+^ (% live cells) cells compared to IgG2a-control. BEV treatment led to significantly increased levels of CD4^+^GzmB^+^ (% live cells) cells compared to DEXA. Data presented as mean ± SEM. Statistical significance determined by one-way ANOVA (*P <.05, **P <.01) (B) FC histograms showing Granyzme B (GzmB) expression in CD8^+^ and CD4^+^ T cells isolated from *ex vivo* co-culture experiment treatment groups. BEV treatment resulted in the highest median fluorescence intensity of GzmB, indicating improved cytotoxic function. (C) *Ex vivo* tumor cell proliferation assay. Spleenocytes co-cultured with SMA560 cells under BEV-treatment conditions significantly suppressed tumor cell proliferation compared to DEXA-treatment and IgG-control. Statistical significance was determined by one-way ANOVA (*P < .05, **P <.01).

Flow cytometry analysis showed significantly higher expression of the inhibitory checkpoint molecule Programmed death 1 (PD-1) on CD4^+^ cells in DEXA-treated tumors, compared to IgG2a-treated controls (Figure 5A, Supplementary Figure S7). BEV-treated brains did not exhibit altered PD-1 expression on CD4^+^ or CD8^+^ cells as compared to control groups (Figure 5A, Supplementary Figure S8C). However, tumors from BEV-treated mice exhibited significantly higher proportion of T cell activation marker GzmB in CD4^+^ cells as compared to NaCl- and DEXA-treated animals (Figure 5A). No significant differences regarding GzmB expression were observed in CD8^+^ subsets (Supplementary Figure S8C).

### SMA560-VM/Dk Co-Culture Models *Ex Vivo* Reveal Immunomodulation of T Cells and Enhanced Tumor Cell Killing

*Ex vivo* co-culture experiments of glioma long-term cell line SMA560 and autologous, stimulated spleenocytes from VM/Dk mice showed immunomodulatory effects of subtoxic DEXA and BEV treatment. Under BEV treatment, spleenocytes significantly suppressed SMA560 tumor cell proliferation compared to DEXA- and IgG-control treatment conditions (**P < .01, one-way ANOVA; Figure 5C). Flow cytometry analysis revealed highest median fluorescence intensity of GzmB in CD4^+^ T cells under BEV treatment compared to DEXA- and saline-control treatment (*P < .05; Figure 5B, Supplementary Figure S9A). Among CD8^+^ T cells, median fluorescence intensity of GzmB was significantly higher in BEV- compared to DEXA-treated conditions (*P < .05; Figure 5B).

## Discussion

DEXA and BEV are widely used to control tumor- and therapy-associated edema, yet their molecular effects on glioblastoma metabolism and immunity are still not fully understood. Here, we demonstrate that DEXA, but not BEV, induces marked metabolic and immunologic alterations in glioblastoma.

DEXA-treated tumor tissue exhibited elevated lactate and reduced glucose, consistent with a shift toward a tumor-supportive metabolic state and increased aerobic glycolysis (Figure 1B and C). Alterations in homocysteine, cystathionine, and 2-hydroxybutyrate indicate enhanced transsulfuration flux for the production of glutathione, reflecting increased oxidative stress. Together with elevated taurine and hypoxanthine, these data reveal substantial metabolic dysregulation and a higher level of aerobic glycolysis (Warburg effect) in DEXA-treated glioblastoma samples, illustrated by consumed glucose and accumulation of lactate, which itself recently was found to be actively involved in epigenetic regulations and pH-dependent acidification.^20-23^ As all patients received perioperative DEXA, potential acute effects of corticosteroid use are effectively controlled for and can be considered part of the baseline condition in our analyses. Furthermore, as glucocorticoids primarily act via long-term transcriptional regulation, the observed differences are more likely linked to sustained treatment exposure rather than short-term administration.

Metabolic reprogramming is a key determinant of immune function within the tumor microenvironment.^24^^,^^25^ Due to the retrospective nature of this study, patient-matched primary cell cultures or patient-derived xenograft models were not available, necessitating the use of established glioma long-term cell lines. DEXA altered cytokine secretion in LN229 cells *in vitro*, reducing IL6 and IL7 while increasing IL8 (Figure 2B), thus diminishing T cell-supportive signaling. Of note, this cytokine pattern was mirrored in DEXA-treated murine tumors (Figure 3C) and in DEXA-exposed glioblastoma patient samples, which exhibited elevated CXCL1, IL8, and CSF1 (Table 1, Supplementary Figure S3). Metabolic reprogramming enables tumor cells to thrive in hypoxic and nutrient-deprived environments while concurrently shaping the immune landscape.^26^ The accumulation of metabolites such as lactate, kynurenine, and adenosine contributes to a hostile environment for effector immune cells, promoting the recruitment and activity of immunosuppressive populations, including T_regs_ and glioma-associated macrophages and microglia (GAMMs).^27-31^ The release of IL8 - a potent chemoattractant for neutrophils - combined with the observed reduction in IL7 secretion, is suggestive of suppression of cytokine signaling important for T cell proliferation and survival while promoting a more immunosuppressive, myeloid-skewed environment.^32-34^ We conclude that DEXA contributes to shaping the glioma microenvironment toward a more myeloid-permissive state through both metabolic and cytokine-mediated pathways.^34^^,^^35^

*In vivo* immune profiling demonstrated that DEXA reduced intratumoral T cells while expanding FOXP3^+^ regulatory T cell subsets (CD4^+^FOXP3^+^ and CD8^+^FOXP3^+^) (Figure 3C and D). Increased PD-1 expression on CD4^+^ T cells in DEXA-treated tumors (Figure 5A) indicates T cell exhaustion.^36^ Lower CD4^+^ counts have been reported under DEXA in high-grade glioma patients.^6^ In contrast, BEV induced minimal metabolic shifts and preserved a more immune-supportive cytokine milieu (Supplementary Figure S5). Patient-derived data following BEV treatment were not available, as its administration is restricted to progressive tumor settings and patients are not treated with BEV prior to tumor resection. This represents an inherent limitation and highlights the need for potential future studies incorporating such cohorts of BEV-treated patients. However, BEV-treated LN229 cells upregulated CCL5, IL7, and CXCL chemokines (Figure 2A), and BEV-treated mice exhibited increased CD4^+^GzmB^+^ effector T cells and reduced FOXP3^+^ T_regs_ (Figures 3D and 5A, Supplementary Figures S7 and S8). These data suggest that BEV relieves edema through VEGF inhibition without compromising antitumor immunity or promoting tumor-supportive metabolic adaptations. While these findings were obtained in a murine model, concordant metabolic and cytokine alterations observed in DEXA-exposed patient samples support their relevance to human glioma biology. The SMA560-VM/Dk syngeneic glioma model was selected to enable investigation in an immunocompetent setting while maintaining moderate immunogenicity compared to chemically induced models, complementing our *in vitro* and patient-derived approaches under non-cytotoxic treatment conditions. Both vascularization and baseline hypoxia were confirmed in the SMA560-VM/Dk model by CD31 and CA IX staining, respectively (Supplementary Figure S10), supporting its biological relevance. Nevertheless, differences between murine glioma models and human disease remain an inherent limitation.

DEXA can exert cytostatic effects *in vitro*, and an anti-proliferative effect of DEXA has previously been demonstrated in several cell glioma lines (eg T98G, A172, 86HG39).^37^^ , ^^38^ Still, these observations must be weighed against its profound immunosuppressive activity *in vivo*. Concentrations required to inhibit glioma proliferation *in vitro* often exceed physiologically achievable intratumoral levels, and our own *in vivo* model (SMA560) showed no DEXA-induced cytotoxicity at doses of up to 200 µM (Supplementary Figure S4G). BEV on the other hand, does not impair tumor cell viability or overall tumor growth rates *in vitro* and *in vivo* (Figure 4A, Supplementary Figure S4H).^39^^,^^40^

Collectively, our data show that DEXA induces metabolic and immunologic changes consistent with reduced antitumor immune activity, while BEV spares both tumor metabolism and immune function. Clinical data from glioblastoma immunotherapy trials suggest that concurrent corticosteroid exposure is associated with inferior outcomes. In particular, subgroup analyses of the CheckMate 143 trial showed decreased median OS in patients with baseline corticosteroid use in the nivolumab (NIVO, anti-PD-1) arm.^12^ On the other hand, BEV has not consistently improved OS despite demonstrated benefits in edema control and PFS.^13^^,^^41^ Clinical experience with the combination of NIVO and BEV remains limited to early-phase studies. For example, the randomized phase II NAVAL trial (NCT03452579) reported comparable PFS and OS between standard- and low-dose BEV in combination with NIVO, with a post hoc survival benefit observed in elderly patients (≥ 60 yrs) receiving standard-dose BEV.^42^ Given the growing integration of immunotherapy into glioblastoma treatment, these results underscore the need for careful, indication-specific use of glucocorticoids and support further evaluation of BEV as a steroid-sparing strategy for edema control in future clinical trials.

## Supplementary Material

vdag147_Supplementary_Data

## Data Availability

Metabolomics data have been deposited to the EMBL-EBI MetaboLights database (DOI: 10.1093/nar/gkz1019, PMID : 31691833) with the identifier MTBLS3873. The complete data set can be accessed at https://www.ebi.ac.uk/metabolights/MTBLS3873.^15^
