# Supplementary material for: Differential modulation of glioma metabolism and the tumor microenvironment following dexamethasone and bevacizumab treatment
Source: Neurooncol Adv. 2026 Jun 8;8(1):vdag147. doi: 10.1093/noajnl/vdag147 (PMC13385348; doi:10.1093/noajnl/vdag147)
Supplement: vdag147_Supplementary_Data [file vdag147_supplementary_data.docx]

**Differential alterations in glioma metabolism and the tumor microenvironment following dexamethasone and bevacizumab treatment**

Louise Maise^1^, Felix Krautwurst^1^, Surender Surender^1^, Gyuntae Bae^2,3^, Laimdota Zizmare^2,3^, Jil Trampert^1^, Marko Maric^1^, Susanne Beck^1,3,4^, Marcos Tatagiba^4,5^, Christoph Trautwein^2,3,6,7^, Hannes Becker^1,5^, Ghazaleh Tabatabai^1,3,4^

**Supplementary Information**

**Contents**

- Supplementary Materials

- Supplementary Figures

- Supplementary Tables

**Supplementary Materials**

**List of Antibodies**

| **Application** | **Description** | **Catalog-No.** | **Manufacturer** | **Manufacturer Location** |
| --- | --- | --- | --- | --- |
| Immunohistochemistry (IHC) | CD3 | ab16669 | Abcam | Cambridge, UK |
| IHC | CD4 | ab183685 | Abcam | Cambridge, UK |
| IHC | CD8 | ab22378 | Abcam | Cambridge, UK |
| IHC | CD11b | ab133357 | Abcam | Cambridge, UK |
| IHC | CD31 | 550274 | BD Biosciences | San Jose, CA, USA |
| IHC | Carbonic Anhydrase (CA) IX | AF2344 | R&D Systems | Minneapolis, MN, USA |
| IHC Secondary | Biotinylated Horse anti-rabbit | BA-1100 | Vector Laboratories | Burlingame, CA, US |
| IHC Secondary | Biotinylated Goat anti-rat | BA-9401 | Vector Laboratories | Burlingame, CA, US |
| Flow Cytometry (FC) | CD4 APC anti-mouse | AB_2563024 | BioLegend | San Diego, CA, US |
| FC | MHC-II APC-Cy7 anti-mouse | AB_1659252 | BioLegend | San Diego, CA, US |
| FC | CD3 APC Fire 750 anti-mouse | AB_2629686 | BioLegend | San Diego, CA, US |
| FC | FOXP3 APC anti-mouse | REA788 | Miltenyi Biotec | Bergisch Gladbach, DE |
| FC | CD8A BV510 anti-mouse | AB_2561389 | BioLegend | San Diego, CA, US |
| FC | Granzyme B FITC anti-human/mouse | AB_2687029 | BioLegend | San Diego, CA, US |
| FC | CD4 FITC anti-mouse | AB_1279237 | BioLegend | San Diego, CA, US |
| FC | Viability Dye eFluor 450 | 65-0863-18 | Thermo Fisher Scientific | Waltham, MA, US |
| FC | PD-L1 PE anti-mouse | AB_2894673 | BioLegend | San Diego, CA, US |
| FC | CD45 PE-Cy7 anti-mouse | 552848 | BD Biosciences | San Jose, CA, USA |
| FC | CD25 PE-Cy5 anti-mouse | AB_312859 | BioLegend | San Diego, CA, US |
| FC | CD279 PE-Cy5 anti-mouse | AB_2910294 | BioLegend | San Diego, CA, US |

**Supplementary Figures**


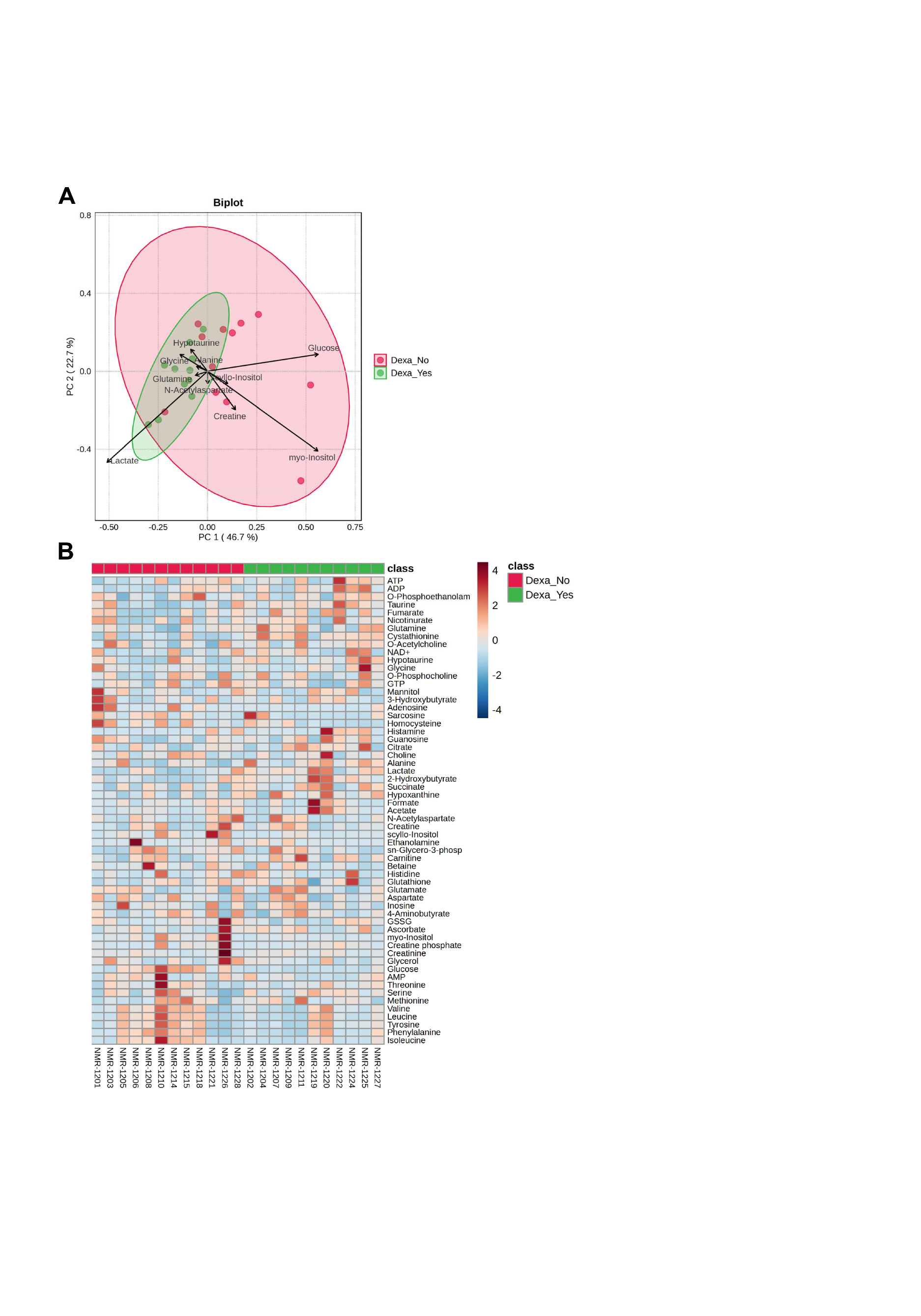


**Supplementary Figure S1**

**Metabolomics profiling of glioma patient tumor tissue samples by ^1^H-NMR spectroscopy highlights DEXA-associated metabolic alterations.** (A) Principal component analysis (PCA) scores plot demonstrating robust separation between DEXA-treated and steroid-naive glioblastoma. (B) Heatmap showing normalized concentration of each metabolite in the individual samples.

**Alt text:** Two-panel supplementary figure. (A) shows a PCA plot with DEXA-treated samples separated from naïve samples. (B) shows a heatmap of metabolite concentrations across individual samples.


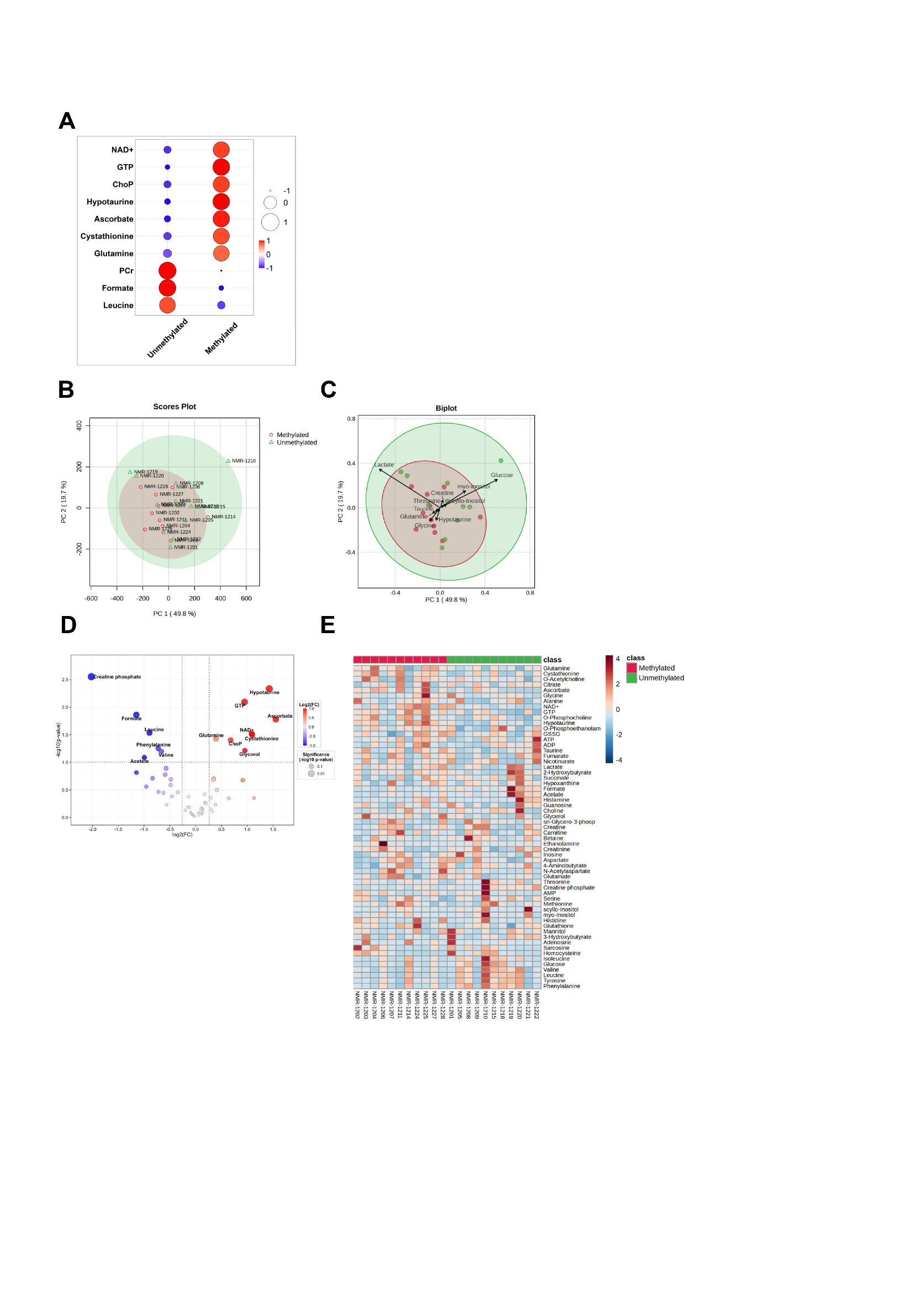


**Supplementary Figure S2**

**Metabolomic profiling of glioma patient tumor tissue samples by ^1^H-NMR** **spectroscopy highlights methylation status-associated metabolic alterations.** ChoP, Phosphocholine. PCr, Phosphocreatine. (A) Bubble plots illustrating metabolites with differential metabolite abundance between methylated and unmethylated patient samples. Size represents normalized average difference between groups (B, C) Principal component analysis (PCA) scores plot demonstrating robust separation between unmethylated and methylated glioblastoma patient samples (D) Volcano plot illustrating significant metabolic changes, with 10 metabolites (NAD+, GTP, o-phosphocholine, hypotaurine, ascorbate, cystathionine, glutamine, leucine, formate, and phosphocreatine) showing P < 0.1 and fold change > 1.2 in methylated versus unmethylated patient samples. (E) Heatmap showing normalized concentration of each metabolite in the individual samples

**Alt text:** Multi-panel figure on MGMT methylation metabolomics with subfigures A-E. (A) shows bubble plots of different metabolites between unmethylated and methylated samples. (B) and (C) show PCA separating unmethylated from methylated samples. (D) shows a volcano plot highlighting 10 significantly altered metabolites. (E) shows a clustered heatmap of metabolite concentrations across samples.


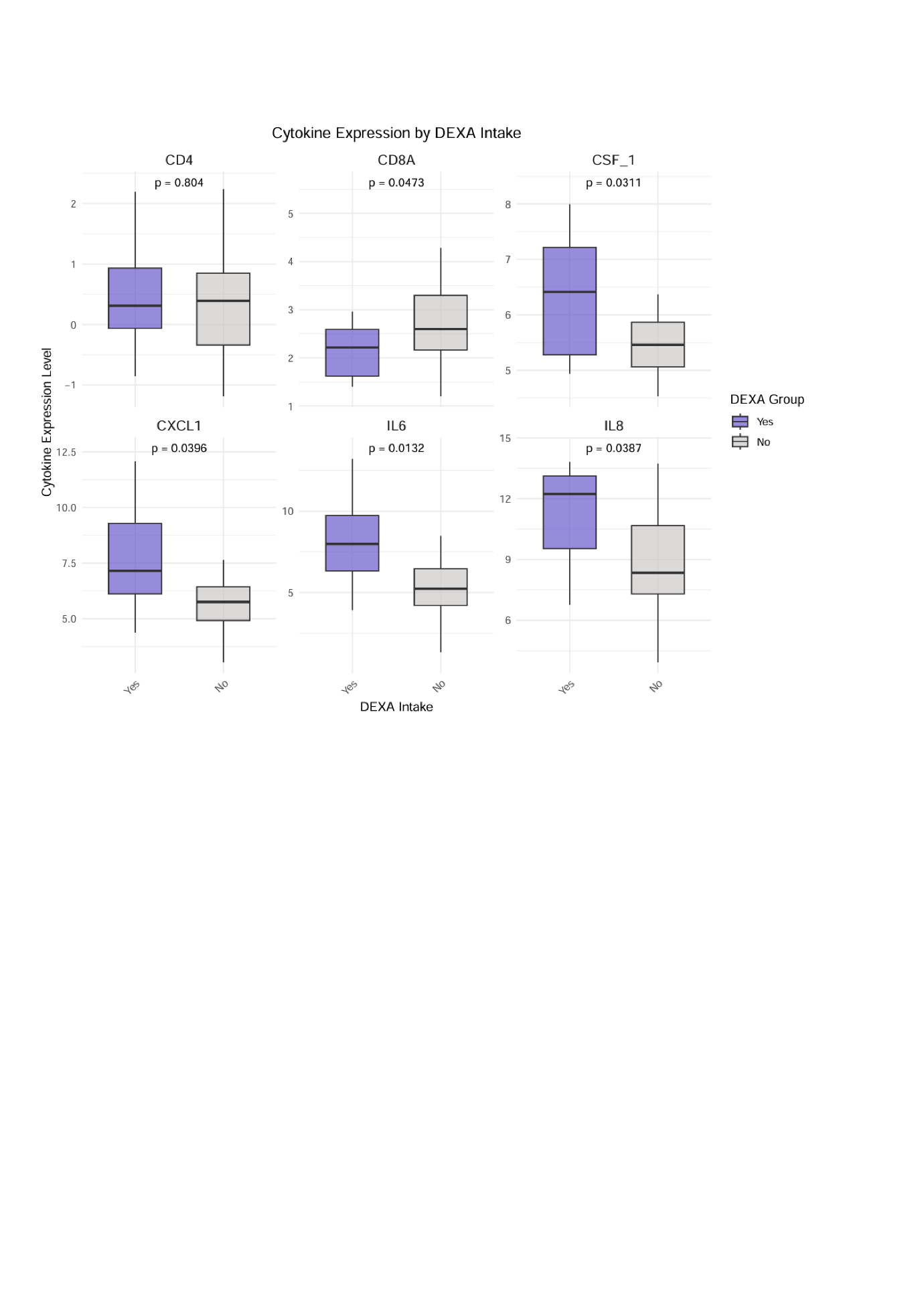


**Supplementary Figure S3**

**Cytokine expression levels stratified by DEXA intake**. Boxplots depict median and interquartile range of expression levels of selected cytokines and immunological markers (CD4, CD8A, CSF1, CXCL1, IL6, and IL8) in patients with (purple) or without (grey) DEXA intake. Statistical significance was assessed using unpaired *t*-tests, with corresponding P values displayed above each panel. Cytokines with significant differences (P < 0.05) between treatment groups include CD8A, CSF1, CXCL1, IL6, and IL8, all showing higher expression levels in the DEXA intake group.

**Alt text:** Six box plots of cytokine/marker levels for CD4, CD8A, CSF1, CXCL1, IL6 and IL8 in patient samples stratified by DEXA intake and P-values labeled above.

**
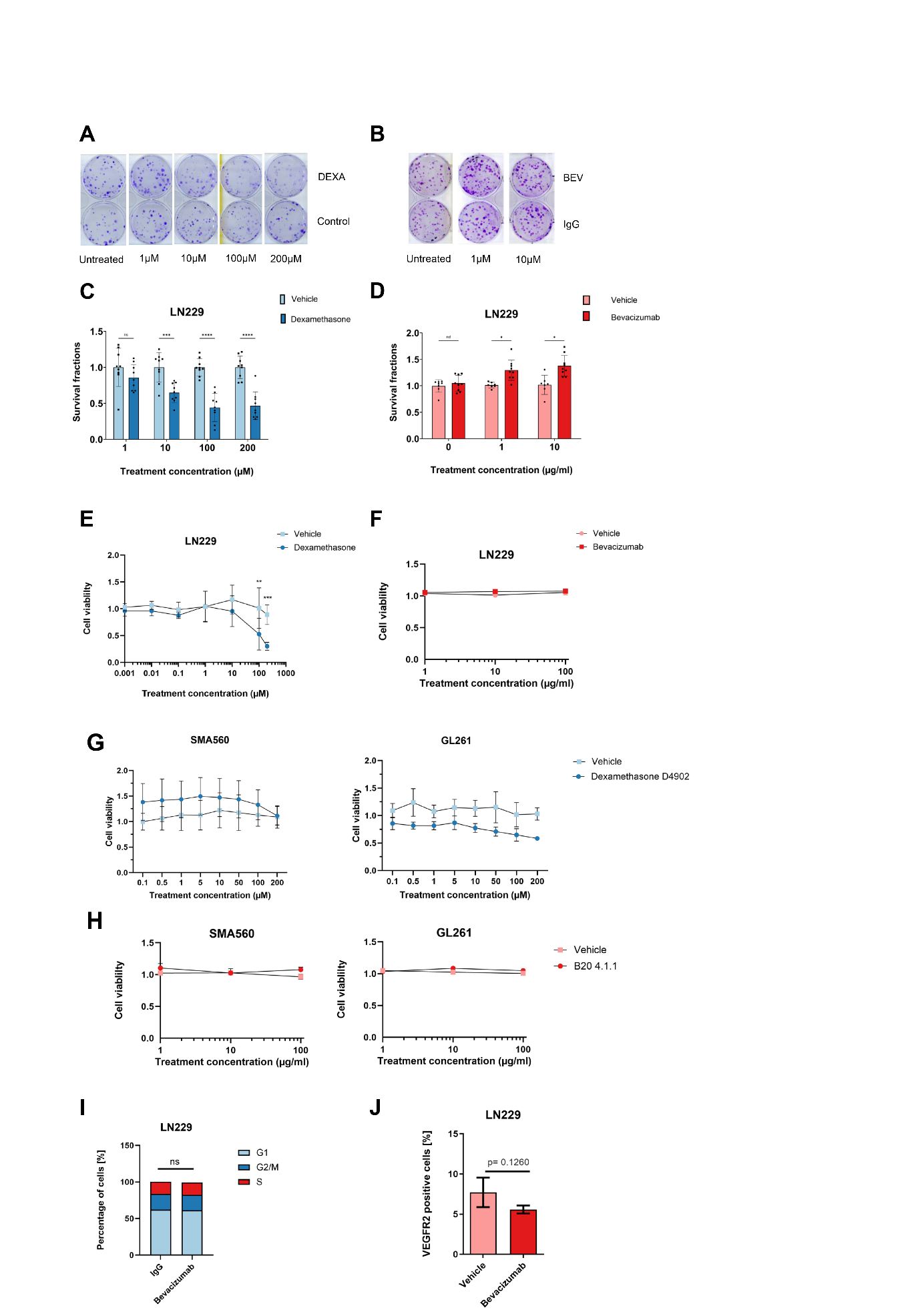
**

**Supplementary Figure S4**

**Cell culture in LN229 cells reveals distinct effects of BEV and DEXA *in vitro.*** (A, B) Representative images of clonogenic survival assays of LN229 cells treated with either DEXA (A) or BEV (B). (C, D) Quantification of clonogenic survival fractions for LN229 cells treated with increasing concentrations of DEXA (C) or BEV (D). (E, F) Cell viability assays showing dose–response curves for DEXA (E) and BEV (F) in LN229 cells. (G) Cell viability assays showing dose–response curves for DEXA in SMA560 and GL261 cells. (H) Cell viability assays showing dose–response curves for BEV in SMA560 and GL261 cells. (I) Cell cycle distribution analysis of LN229 cells treated with BEV or vehicle control showing percentages in G1, G2/M, and S phases. (J) Flow cytometry analysis of VEGFR2-positive cells in LN229 treated with BEV or vehicle control. Data are presented as mean ± SEM; ns, not significant. Statistical analysis was done using multiple t-tests (* P < 0.05, ** P< 0.01, *** P < 0.001, **** P < 0.0001).

**Alt text:** Multi-panel figure with subfigures labeled A-J. (A-D) show pictures of representative wells and bar graphs with results of clonogenic survival assays in LN229 cells with DEXA dose-dependent colony reduction but minimal BEV effect. (E-H) show dose-response curves with dose dependent-reduction in cell viability under DEXA but minimal BEV effect in LN229, SMA560 and GL261 cells. (I) shows graph of cell cycle analysis without significant changes under BEV treatment. (J) shows bar graphs of VEGFR2 signal in flow cytometry analysis of LN229 cells with reduced signal under BEV treatment.


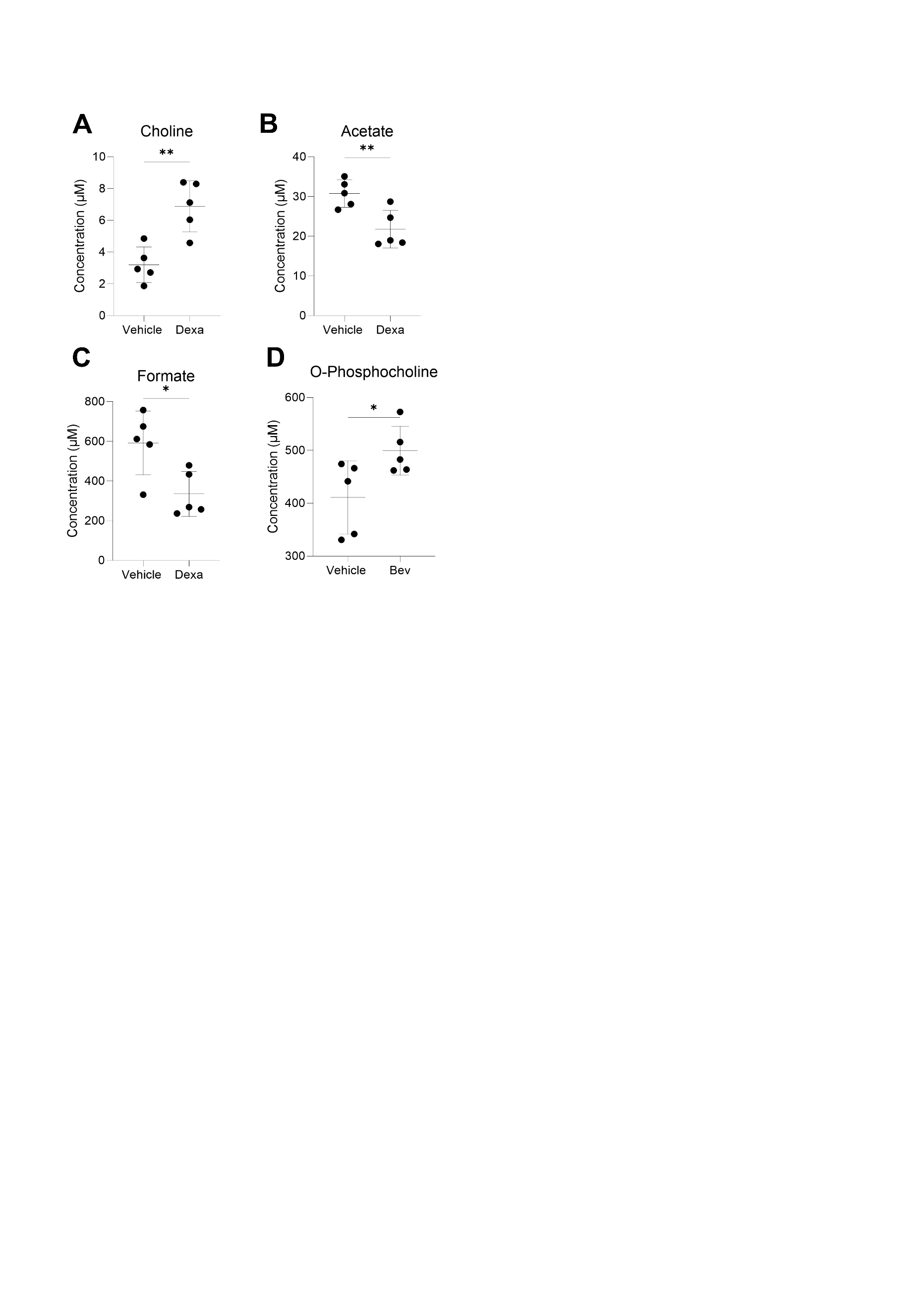


**Supplementary Figure S5**

**DEXA-dependent changes of metabolites alter energy and cell growth metabolism *in vitro***. ^1^H-NMR spectroscopy of cell culture extracts reveals DEXA- and BEV-modulated metabolites in LN229 glioma cells. (A-C) Dot-boxplots showing metabolomics analysis of LN229 cells treated with DEXA (D4902) or vehicle control for 24 h, revealing significant increases in choline (A, **P < 0.01) and acetate (B, **P < 0.01) concentrations, and a significant decrease in formate (C, *P < 0.05) compared to vehicle. (D) Dot-boxplots showing O-phosphocholine concentration in LN229 cells treated with BEV (HYP-P9906) or vehicle control for 24 h, with a significant increase (*P < 0.05) following BEV treatment. Data is presented as median with interquartile range (IQR), statistical significance was determined by unpaired t-test (*P < 0.05, **P < 0.01).

**Alt text:** Four dot-box plots (labelled A-D) of metabolite concentrations in LN229 cells. (A-C) show higher choline/acetate and lower formate after DEXA versus vehicle control. (D) shows higher O-phosphocholine after BEV versus IgG control.

**
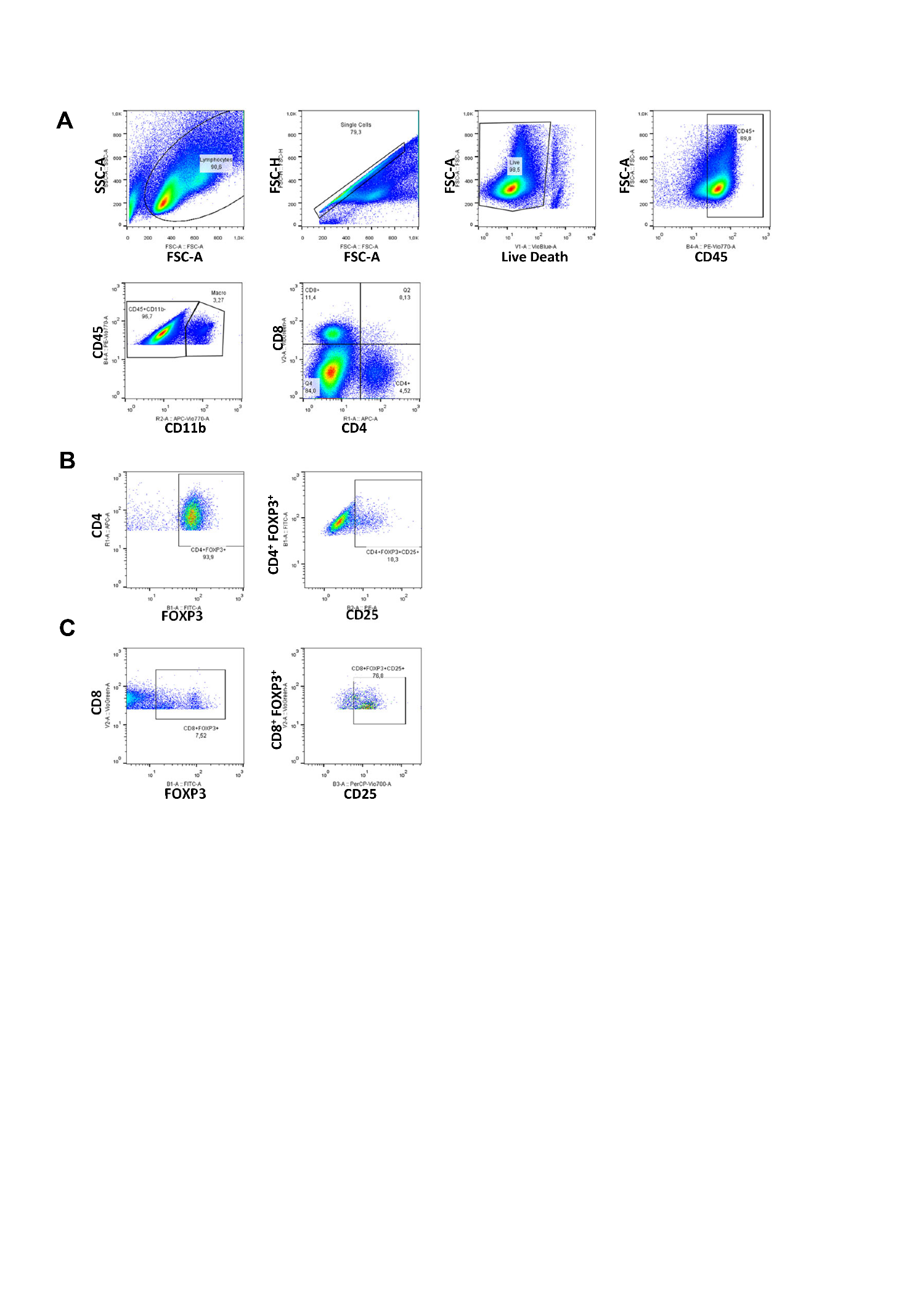
**

**Supplementary Figure S6**

***In vitro* and *in vivo* gating strategies for flow cytometry.** (A) Representative gating strategy for identification of cell cycle state of glioma long-term cell line after treatment. (B) Representative gating strategy for identification of live, single and immune cells from murine tumor tissue. Lymphocytes were first gated based on forward scatter (FSC) and side scatter (SSC) profiles. Singlet cells were selected using FSC-A versus FSC-H, and live cells were identified using a viability dye. CD45⁺ immune cells were then gated from the live singlet population. CD45⁺ cells were further gated for CD11b expression to identify macrophages (CD45⁺CD11b⁺). CD45⁺ T cells were then analyzed for CD4 and CD8 expression to distinguish between CD4⁺ and CD8⁺ T cell populations. (C, D) Regulatory T cell analysis. CD4⁺ (C) and CD8^+^ (D) T cells were assessed for Forkhead box P3 (FOXP3) expression to identify total T_regs_ (CD4⁺FOXP3⁺/ CD8^+^FOXP3^+^), and further gated for CD25 expression to define activated T_regs_ (CD4⁺FOXP3⁺CD25⁺/ CD8^+^FOXP3^+^CD25^+^).

**Alt text:** Multi-panel figure with subfigures A-C showing flow cytometry gating schemes. (A) shows gating of live/single/CD45+/CD11b+/CD4+/CD8+ cells extracted from tumors. (B) shows gating of FOXP3+/CD25+ regulatory T cells within CD4+ compartment. (C) shows gating of FOXP3+/CD25+ regulatory T cells within CD8+ compartment.

**
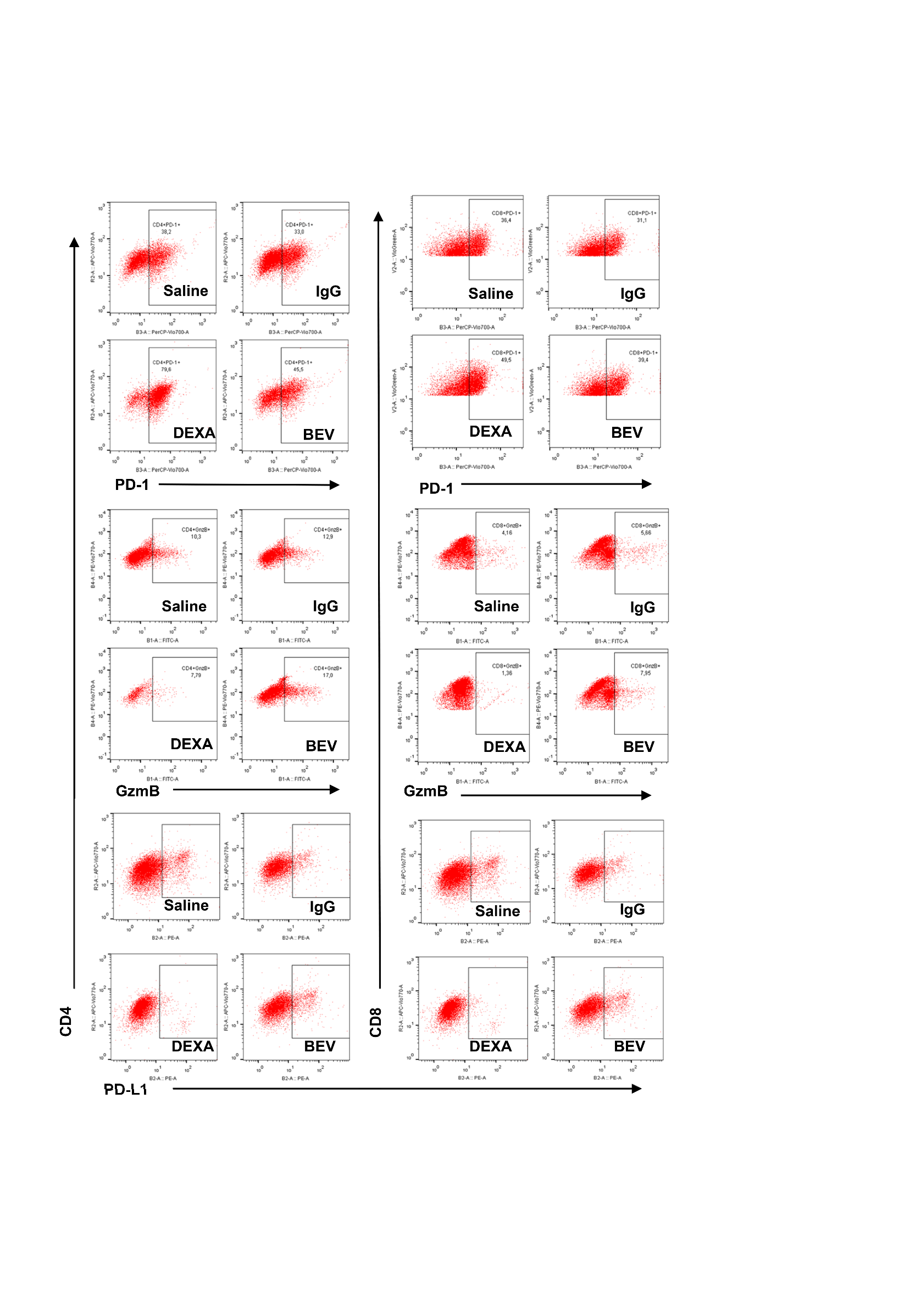
Supplementary Figure S7**

**Representative flow cytometry plots showing the gating strategy used to assess Programmed cell death protein 1 (PD-1), Granzyme B (GzmB), and Programmed death-ligand 1 (PD-L1) expression in tumor-infiltrating CD4⁺ and CD8⁺ T cells isolated from murine tumors treated with Saline, IgG control, DEXA, or BEV**. The left column of each panel shows gating on CD4⁺ T cells, while the right column shows gating on CD8⁺ T cells. The top two rows display the frequency of PD1⁺ cells, the middle two rows show GzmB^+^ cells, and the bottom two rows depict PD-L1⁺ cells within each T cell subset. Percentages indicate the proportion of marker-positive cells within the respective CD4⁺ or CD8⁺ T cell populations. This gating strategy was applied consistently across all experimental groups.

**Alt text:** 6×4 grid of flow density plots for PD-1/GzmB/PD-L1 in CD4+ (left two rows)/CD8+ (right two rows) T cells across saline-, IgG-, DEXA- and BEV-treated tumors, with % positive cells in respective gates varying by treatment and marker.

**
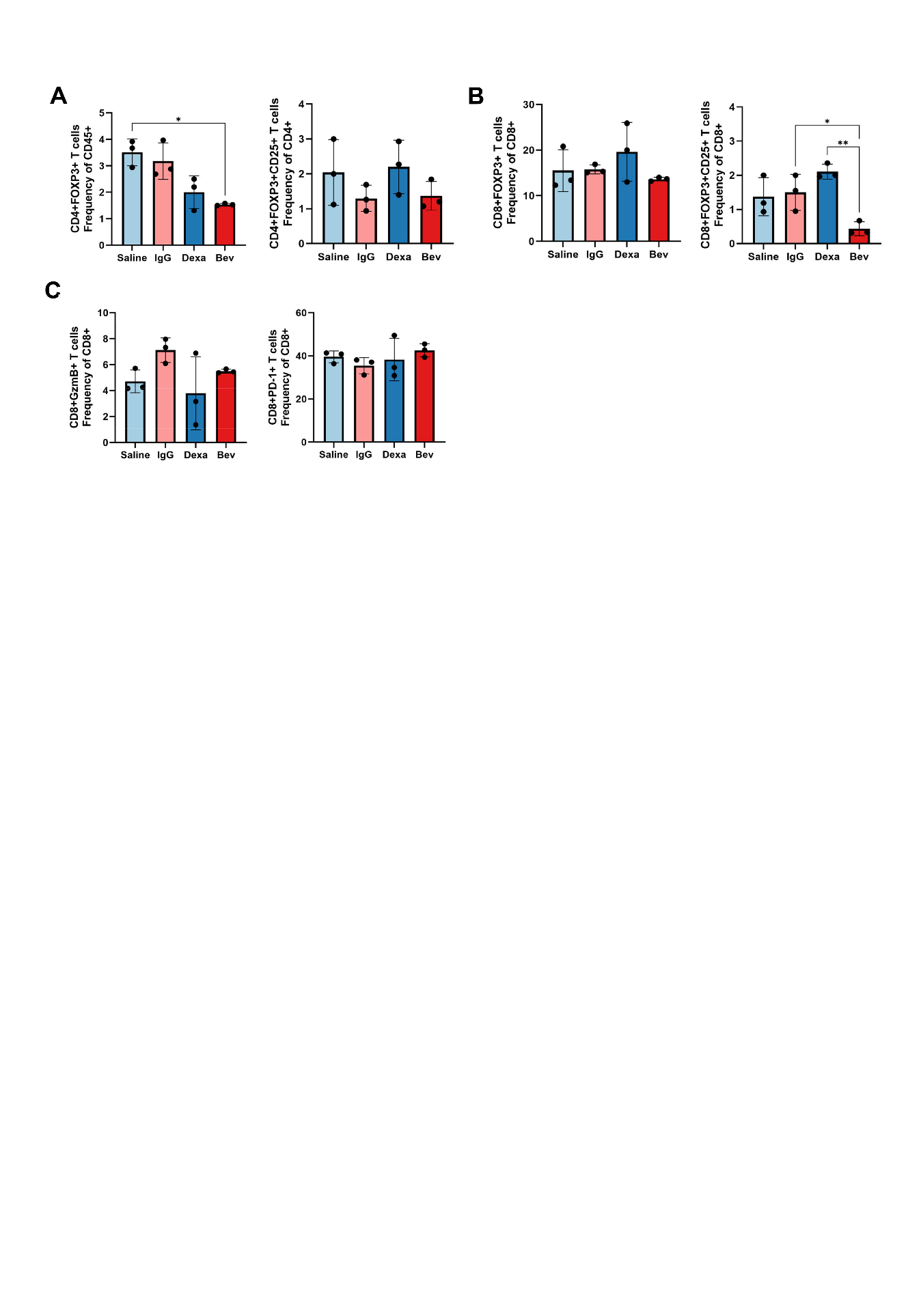
**

**Supplementary Figure S8**

**Flow cytometry analysis of tumor-infiltrating lymphocytes in murine tumors following different treatments.** Single-cell suspensions were analyzed by flow cytometry to assess the frequency and phenotype of tumor-infiltrating CD4⁺ and CD8⁺ T cells. (A) Quantification of CD4⁺FOXP3⁺ regulatory T cells and CD4⁺FOXP3⁺CD25⁺ T cells. (B) Quantification of CD8⁺FOXP3⁺ T cells and CD8⁺FOXP3⁺CD25⁺ T cells. (C) Quantification of GzmB^+^ and PD1^+^ CD8^+^ cells. Data are presented as mean ± SEM. Statistical significance was determined by one-way ANOVA followed by Tukey’s multiple comparisons test (*P < 0.05, **P < 0.01).

**Alt text:** Three-panel figure with subfigures A-C showing bar graphs of flow cytometry data across saline-, IgG-, DEXA- and BEV-treated tumors with results of statistical analysis. (A) shows CD4+FOXP3+/CD25+ regulatory T cell frequencies with lowest regulatory T cell (CD4+FOXP3+) frequency under BEV treatment. (B) shows CD8+ FOXP3+/CD25+ T cell frequencies with lowest regulatory T cell (CD8+FOXP3+CD25+) frequency under BEV treatment. (C) shows CD8+ GzmB+/PD-1+ cell frequencies with no significant differences across treatment groups.

**
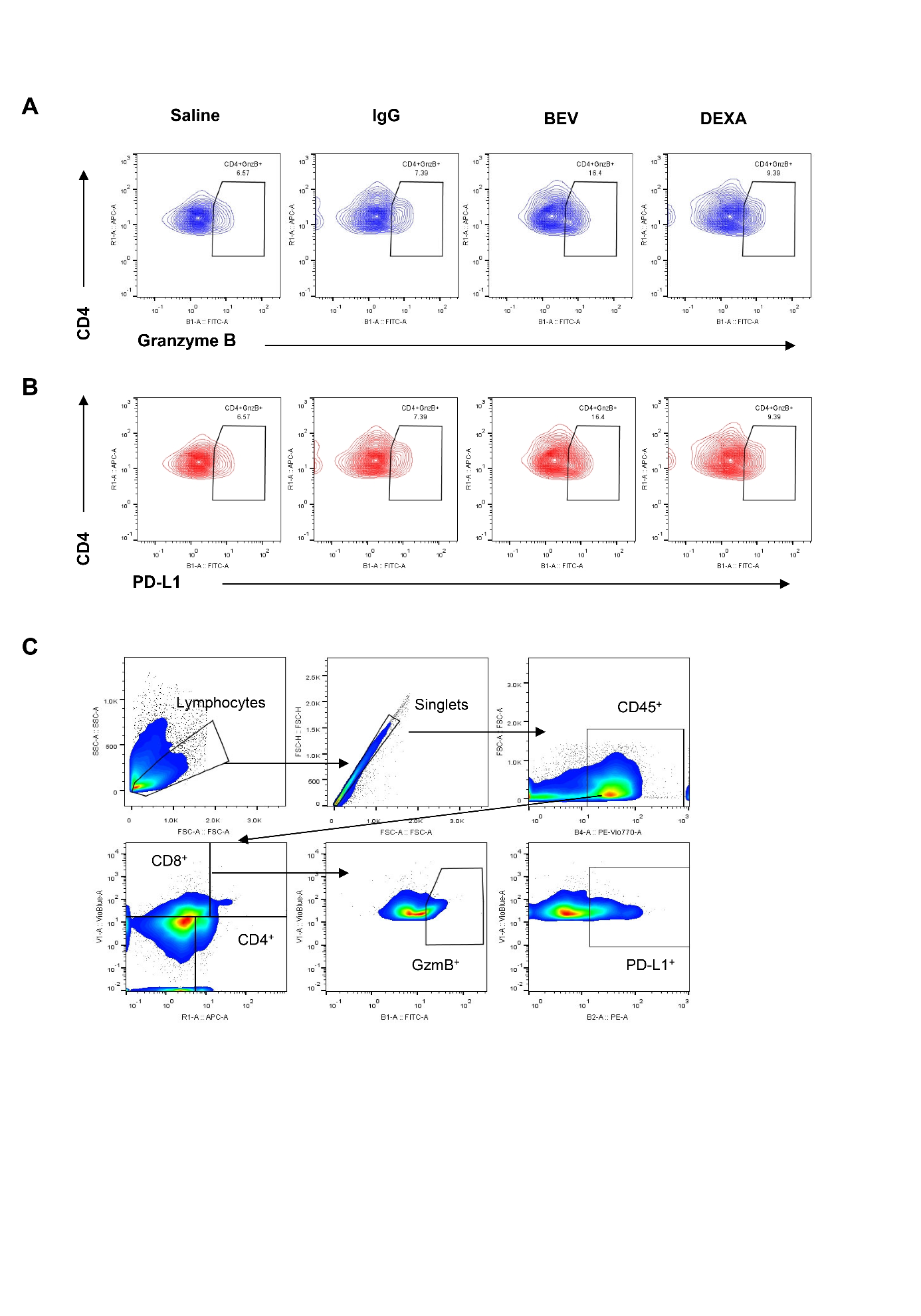
**

**Supplementary Figure S9**

**Gating strategy and representative flow cytometry plots for *ex vivo* analysis of tumor-infiltrating immune cells.** (A) Representative gating of GzmB⁺CD4⁺ T cells across treatment groups. (B) Representative gating of PD-L1⁺CD4⁺ T cells across treatment groups. (C) Schematic of the gating strategy used. Lymphocytes were first identified based on FSC-A versus SSC-A properties, followed by singlet discrimination using FSC-A versus FSC-H to exclude doublets. Live CD45⁺ leukocytes were then gated to define immune cell populations. Subsequent gates distinguished CD4⁺ and CD8⁺ T cells, followed by assessment of functional and phenotypic markers such as GzmB and PD-L1. This gating strategy was applied uniformly across all groups to ensure consistent and accurate quantification of tumor-infiltrating immune cell subsets.

**Alt text:** Multi-panel figure with subfigures A-C. (A) shows representative flow plots for GzmB+ gates in CD4+ T cells across saline, IgG, DEXA and BEV-treatment groups. (B) shows according PD-L1+ gates in CD4+ T cells. (C) shows sequential gating strategy: lymphocyte/ singlet/ live/ CD45+/ CD4+/ PD-1 or Granzyme B.


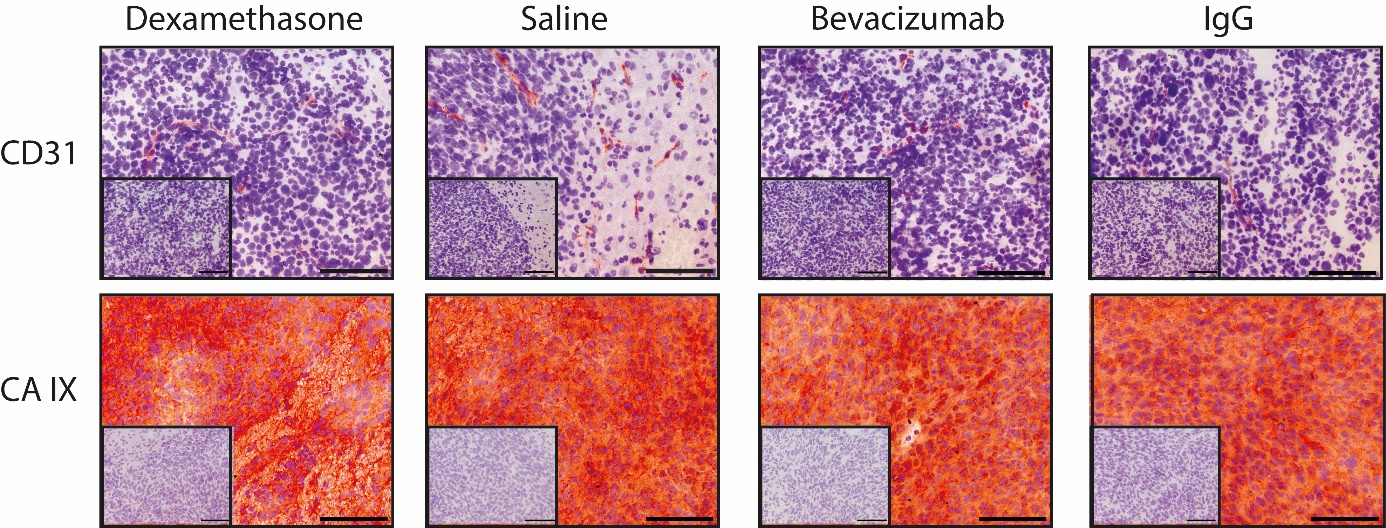


**Supplementary Figure S10**

**Immunohistochemical staining for tumor-associated vasculature (CD31) and long-term hypoxia marker Carboanhydrase (CA) IX.** Representative images of SMA560 glioma tumors from VM/Dk mice treated with dexamethasone, saline, bevacizumab or IgG control stained by immunohistochemistry for CD31 (top row) and CA IX (bottom row). Insets show secondary antibody control-stained tissue, 200x magnification, scalebars 100 µm.

**Alt text:** 2x4 panel of microscopy images with scalebars showing immunohistochemical staining of murine brain tissue from DEXA, saline, BEV and IgG treatment groups for markers CD31 and CA IX with insets showing no positive staining in secondary antibody control-stained tissue.

**Supplementary Tables**

**Supplementary Table S1**: **Baseline Characteristics of Glioblastoma Patients Stratified by DEXA Intake**

|  |  | **DEXA intake** | |  |
| --- | --- | --- | --- | --- |
| **Characteristic** | **N** | **No, N = 18^A^** | **Yes, N = 12^A^** | **P value^B^** |
| **Mean age at diagnosis (years)** | 30 | 57 (12) | 65 (13) | 0.079 |
| **Age group at diagnosis** | 30 |  |  | 0.073 |
| < 65 yrs |  | 12 (67%) | 4 (33%) |  |
| ≥ 65 yrs |  | 6 (33%) | 8 (67%) |  |
| **Sex** | 30 |  |  | >0.9 |
| Female |  | 4 (22%) | 3 (25%) |  |
| Male |  | 14 (78%) | 9 (75%) |  |
| ***MGMT* promoter methylation status** | 29 |  |  | 0.5 |
| Methylated |  | 8 (47%) | 7 (58%) |  |
| Unmethylated |  | 9 (53%) | 5 (42%) |  |
| Unknown |  | 1 | 0 |  |
| **Tumor localization pre-operative** | 30 |  |  | 0.4 |
| Frontal |  | 4 (22%) | 5 (42%) |  |
| Other |  | 14 (78%) | 7 (58%) |  |
| **KPS pre-operative** | 30 |  |  | >0.9 |
| < 70% |  | 0 (0%) | 0 (0%) |  |
| ≥ 70% |  | 18 (100%) | 12 (100%) |  |

^A^ Mean (SD); n (%). ^B^ Wilcoxon rank-sum test; Pearson’s chi-squared test; Fisher’s exact test. DEXA, Dexamethasone. KPS, Karnofsky Performance Score. This table presents demographic and clinical characteristics of 30 glioblastoma patients, stratified by DEXA exposure (No, n = 18; Yes, n = 12). “Other” tumor localization includes non-frontal sites. “Unknown” indicates missing *MGMT* promoter methylation status. Statistical comparisons of distribution of baseline clinical characteristics between DEXA treatment groups were performed using Wilcoxon rank sum test for continuous variables and Pearson’s Chi-squared test or Fisher’s exact test for categorical variables, with P values indicating significance of differences between DEXA-treated and DEXA-naive groups.

**Supplementary Table S2: Treatment and Clinical Outcome of Glioblastoma Patients Stratified by DEXA Intake**

|  |  | **DEXA intake** | |  |
| --- | --- | --- | --- | --- |
| **Characteristic** | **N** | **No, N = 18^A^** | **Yes, N = 12^A^** | **P value^B^** |
| **Grade of resection** | 30 |  |  | 0.2 |
| Gross total |  | 12 (67%) | 11 (92%) |  |
| Subtotal |  | 6 (33%) | 1 (8.3%) |  |
| **First line of therapy** | 30 |  |  | >0.9 |
| Other |  | 2 (11%) | 0 (0%) |  |
| RT |  | 3 (17%) | 2 (17%) |  |
| TMZ |  | 1 (5.6%) | 1 (8.3%) |  |
| TMZ and RT |  | 12 (67%) | 9 (75%) |  |
| **Overall survival (months)** | 30 |  |  | - |
| Median (IQR) |  | 18 (14, 32) | 20 (12, 23) |  |
| **Progression-free survival (months)** | 30 |  |  | - |
| Median (IQR) |  | 10 (6, 15) | 8 (5, 13) |  |

^A^ Mean (SD); n (%). ^B^ Pearson’s chi-squared test; Fisher’s exact test. DEXA, Dexamethasone. RT, Radiotherapy. TMZ, Temozolomide. “Other” first-line therapy includes treatments other than RT and TMZ. Statistical comparisons of distribution of treatment -associated clinical characteristics between DEXA treatment groups were performed using Pearson’s Chi-squared test or Fisher’s exact test for categorical variables, with P values indicating significance of differences between DEXA-treated and DEXA-naive groups.

**Supplementary Table S3:** **Scoring sheet**

| **Score sheet** | **Evaluation** | **Score** |
| --- | --- | --- |
| **General appearance** |  |  |
| Grooming | smooth, shiny | 0 |
|  | no grooming, dull coat | 1 |
|  | no grooming, dirty | 2 |
|  | no grooming, dirty, piloerection | termination of experiment |
| Eyes | normal | 0 |
|  | slightly sunken, swollen | 1 |
|  | eyelids half-closed | 2 |
|  | severely sunken, eyelids closed | termination of experiment |
| Posture | normal | 0 |
|  | slightly curved | 1 |
|  | arched back | 2 |
|  | severely hunched, legs tucked under the body | termination of experiment |
| Breathing | regular | 0 |
|  | regular, slightly increased | 1 |
|  | clearly increased | 2 |
|  | labored, pumping respiration | termination of experiment |
| Behavior / activity | normal | 0 |
|  | slight change, slowed movement pattern | 1 |
|  | little movement, isolated | 2 |
|  | apathetic, unresponsive | termination of experiment |
| Weight  Relative to baseline weight, corrected for the expected weight gain of same-age, same-sex animals of the same strain during the observation period | normal, continuous gain (±5%) | 0 |
|  | weight loss 5-10% | 1 |
|  | weight loss >10 bis <20% | 2 |
|  | weight loss ≥ 20% | termination of experiment |
|  |  |  |
| **Experiment-related Criteria** |  |  |
| Neurological symptoms | none | 0 |
|  | slight loss of balance, sporadic missteps, mild paresis | 1 |
|  | moderate loss of balance, every third step missed, moderate paresis | 2 |
|  | severe loss of balance, complete inactivity, severe paresis | termination of experiment |
| Pain Assessment – Grimace Scale | normal facial expression | 0 |
|  | 1 pain expression, administer analgesic (Carprofen 5 mg/kg, s.c.) | 2 |
|  | more than 1 pain expression | termination of experiment |
| **Wound Condition** |  |  |
|  | unremarkable | 0 |
|  | slight redness and/or swelling | 1 |
|  | severe redness and/or swelling (Betaisodona solution, daily monitoring) | 2 |
|  | purulent, bleeding wound | termination of experiment |
|  |  |  |
| **Overall Scoring Interpretation** |  |  |
| 0 points | Normal | |
| 1-2 points (additive across categories, no individual score of 2) | Animals checked once daily | |
| 3-4 points (additive across categories or two scores of 2) | Increased monitoring, animals checked twice daily, daily weight, analgesics if needed, wet food; veterinary advice within 24 h if no improvement | |
| ≥ 5 points (additive across categories) | Termination of experiment | |
